# Temporal and genomic analysis of additive genetic variance in breeding programmes

**DOI:** 10.1038/s41437-021-00485-y

**Published:** 2021-12-15

**Authors:** Letícia A. de C. Lara, Ivan Pocrnic, Thiago de P. Oliveira, R. Chris Gaynor, Gregor Gorjanc

**Affiliations:** The Roslin Institute and Royal (Dick) School of Veterinary Studies, The University of Edinburgh, Edinburgh, UK

**Keywords:** Genetic variation, Quantitative trait, Agricultural genetics, Plant breeding, Agriculture

## Abstract

Genetic variance is a central parameter in quantitative genetics and breeding. Assessing changes in genetic variance over time as well as the genome is therefore of high interest. Here, we extend a previously proposed framework for temporal analysis of genetic variance using the pedigree-based model, to a new framework for temporal and genomic analysis of genetic variance using marker-based models. To this end, we describe the theory of partitioning genetic variance into genic variance and within-chromosome and between-chromosome linkage-disequilibrium, and how to estimate these variance components from a marker-based model fitted to observed phenotype and marker data. The new framework involves three steps: (i) fitting a marker-based model to data, (ii) sampling realisations of marker effects from the fitted model and for each sample calculating realisations of genetic values and (iii) calculating the variance of sampled genetic values by time and genome partitions. Analysing time partitions indicates breeding programme sustainability, while analysing genome partitions indicates contributions from chromosomes and chromosome pairs and linkage-disequilibrium. We demonstrate the framework with a simulated breeding programme involving a complex trait. Results show good concordance between simulated and estimated variances, provided that the fitted model is capturing genetic complexity of a trait. We observe a reduction of genetic variance due to selection and drift changing allele frequencies, and due to selection inducing negative linkage-disequilibrium.

## Introduction

This study analyses temporal and genomic trends of additive genetic variance in different stages of a breeding programme. Genetic variance is one of the critical parameters in a breeding programme because it determines the potential for selection (Lush 1937; Falconer and Mackay 1996; Lynch and Walsh 1998; Walsh and Lynch 2018). Estimation of genetic variance has therefore received considerable attention in the literature (Lynch and Walsh 1998; Walsh and Lynch 2018), where most of the attention is on statistical models and approaches for estimation. Surprisingly, far less attention has been given to temporal trends in genetic variance, even though such trends indicate the sustainability of a breeding programme. Recent ability to genotype individuals at scale has renewed interest in analysing genetic variance. This study extends previously proposed framework for temporal analysis of genetic variance using the pedigree-based model of Sorensen et al. (2001), to a new framework for temporal and genomic analysis of genetic variance using marker-based models.

The estimation of genetic variance in breeding programmes has a long history and a recent revival with the advent of genomic information. Historically, the genetic variance was estimated with an analysis of variance (ANOVA) method in optimised experimental designs, ranging from simple parent-offspring or sib groups to North Carolina and diallel designs (Falconer and Mackay 1996; Lynch and Walsh 1998; Bernardo 2002; Awata et al. 2018). With these designs, we partition phenotypic variance into variance between and within groups and ‘translate’ these components into genetic variance based on expected genetic relationships within and between groups. Animal breeders have soon moved from such experimental designs to a general pedigree-based model to analyse their observational data (Henderson 1976). Nearly 30 years later, plant breeders have also adopted the pedigree-based model (Oakey et al. 2006, 2007; Piepho et al. 2008). There were many reasons for this late adoption. One reason is that with the pedigree-based model, we estimate genetic variance between the founders of a pedigree (Sorensen and Kennedy 1984; Kennedy et al. 1988), while genetic variance between their descendants is arguably more relevant for breeding (Piepho et al. 2008). The advent of genomic information revived interest in the estimation of genetic variance and spurred active development of genome-based models (Bernardo 1994, 1996; Meuwissen et al. 2001; VanRaden 2008). The genome-based model replaces expected relationships from the experimental designs or pedigree with realised relationships measured by marker genotypes. The estimate of genetic variance from the genome-based model pertains to genotyped individuals (Hayes et al. 2009) or their relatives (VanRaden 2008). It can be obtained using either a genome-based model with genetic values or with marker effects (marker-based model) (Stranden and Garrick 2009). We note, though, that the resulting ‘genomic variance’ is estimating genetic variance only under certain conditions (Gianola et al. 2009; de los Campos et al. 2015; Rawlik et al. 2020). Specifically, the genome-based model assumes that markers are sufficiently linked to quantitative trait loci (QTL) to capture their effects, and that genetic values at different QTL are uncorrelated. The second assumption about independence will not hold when there are population processes that induce linkage-disequilibrium as a function of QTL (Lynch and Walsh 1998; Walsh and Lynch 2018; Rawlik et al. 2020; Bulmer 1971). We expand on this second assumption in the methods and discussion.

In parallel to the development of data sources and corresponding statistical models, there has been active development in statistical and computational approaches to estimate genetic variance. The three most used are the method of moments, likelihood, and Bayesian approach. The method of moments used with the ANOVA is computationally simple but can yield biased estimates outside of the parameter space. With the likelihood approach, we specify a probability distribution for observed data and find the most likely value of model parameters that would give rise to the observed data (Meyer 1985; Thompson et al. 2005; Thompson 2019). The Bayesian approach improves the likelihood approach in two ways. First, it incorporates prior knowledge for all model parameters (means and variances), improving estimation (Sorensen and Gianola 2007; Hem et al. 2021). Second, it treats all model parameters in a probabilistically consistent manner such that estimation uncertainty is propagated to all estimated model parameters (Sorensen and Gianola 2007). However, the full probabilistic treatment is computationally demanding, despite the availability of sampling methods such as the Monte Carlo Markov Chain (MCMC) (Gilks et al. 1995; Brooks et al. 2011). We can handle this computational issue with an empirical Bayesian approach. In the marker-based model, the empirical Bayesian approach estimates model variances from the data at hand and, conditional on these, estimates all marker effects jointly to account for the uncertainty of estimating marker effects (uncertainty of estimating model variances is ignored) (Sorensen and Gianola 2007; Efron 1996). The full Bayesian approach accounts for uncertainty in estimating model variances and marker effects; however, MCMC on genome-based models with many individuals or markers can be time-consuming. To this end, various dimensionality-reduction approaches have been proposed, for example, singular value decomposition (SVD) of marker genotypes where we fit a small number of principal components that capture a majority of variance in marker genotypes (Tusell et al. 2013; Ødegård et al. 2018).

Variances from pedigree and genome-based models do not inform about temporal and genomic trends in genetic variance because they pertain to a specific group of individuals and encompass the whole genome (Sorensen and Kennedy 1984; Kennedy et al. 1988; Hayes et al. 2009). However, these models can be used for temporal and genomic analyses of genetic variance with some post-processing. Sorensen et al. (2001) showed how to analyse a temporal trend in genetic variance. They fitted a pedigree-based model and inferred genetic variance for several time partitions by sampling realisations of genetic values from the fitted model and calculating the variance of the realisations partitioned in time groups. They used the Bayesian approach and MCMC, but their concept is general and can be used with other statistical and computational approaches. The critical distinction here is between model fitting to estimate statistical/model parameters and post-processing to estimate quantitative genetics parameters. This distinction enables flexibility to fit a generic model, for example, the LASSO (Tibshirani 1996), and to estimate quantitative genetics parameters by post-processing posterior samples or internally within an analysis programme. It also gives a potential to (partially) address issues with the interpretation of estimated ‘genomic variance’ from genome-based models (Gianola et al. 2009; de los Campos et al. 2015; Rawlik et al. 2020). Lehermeier et al. (2017) used the same approach with the marker-based model and analysed the contribution of linkage-disequilibrium to genetic variance. Recently, Allier et al. (2019) also used the marker-based model on data from a maize breeding programme to infer trends in genetic mean and genetic variance as well as the contribution of allele diversity (genic variance) and of linkage-disequilibrium to genetic variance (Lynch and Walsh 1998; Walsh and Lynch 2018; Bulmer 1971).

This work aims to (i) build and validate a flexible framework based on the work of Sorensen et al. (2001), Lehermeier et al. (2017) and Allier et al. (2019), (ii) show how to evaluate the temporal and genomic analysis of additive genetic variance in different stages of a breeding programme, and (iii) indicate population processes that change genome. We also show how different statistical approaches affect the results. To this end, we have validated our work with a simulated breeding programme, used a marker-based model to estimate marker effects and, based on these, estimated temporal and genomic trends in additive genetic variance. The results show that the framework works well and gives valuable insights, provided that the fitted model captures the trait’s genetic complexity.

## Materials and methods

In this section, we present study material and methods in seven parts: (1) simulation of a breeding programme where we generate true genetic values and corresponding variances, and simulated phenotype and marker genotype data, (2) theory for the temporal and genomic analysis of genetic variance assuming we know QTL genotypes and their effects, (3) statistical analysis where we describe marker-based model fitted to the simulated phenotype and marker genotype data, (4) statistical and computational approaches to estimate marker effects, genetic values and variances, (5) validation of the framework with different genetic architectures of a simulated trait, (6) summarising the results and (7) software implementation.

### Breeding programme simulation

**Fig. 1:**
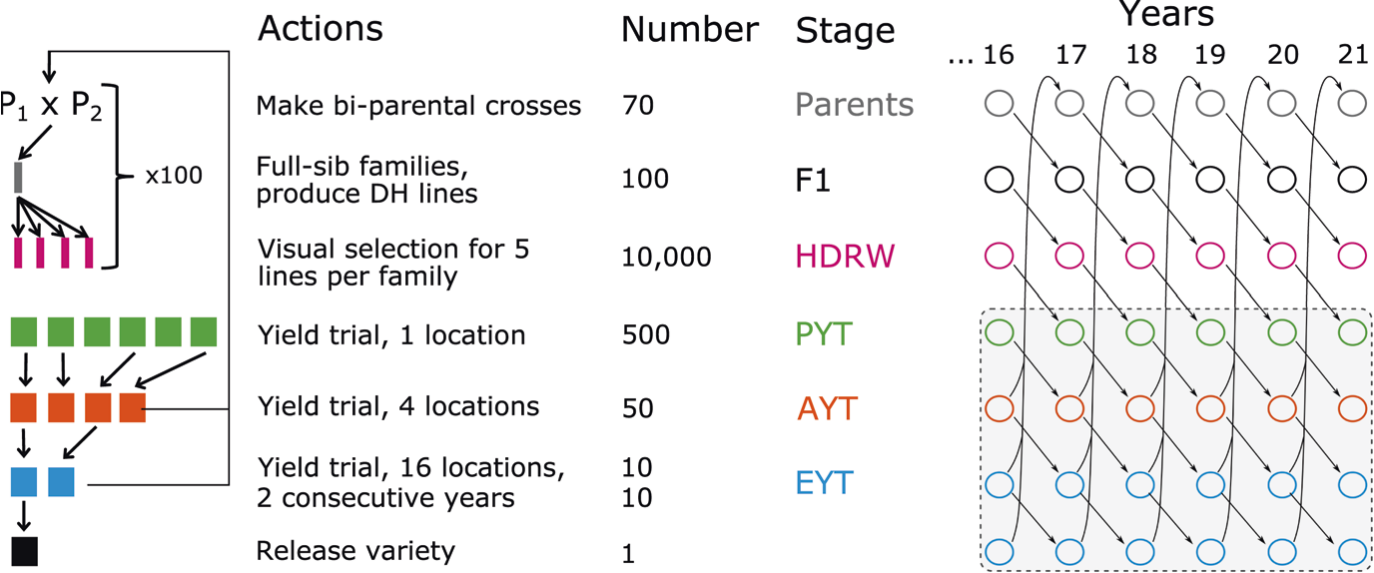
Simulated wheat breeding programme with parents, F1 progeny (F1), headrows (HDRW), preliminary yield trial (PYT), advanced yield trial (AYT), elite yield trial (EYT) and a released variety. The shaded rectangle indicates individuals included in the training population for statistical modelling, from year 16 to year 21.

We simulated an entire wheat breeding programme considering additive genetic architecture for a quantitative trait. We have performed one simulation replicate for most analyses to focus on one dataset, but we also evaluated the consistency of estimates for a subset of analyses on ten simulation replicates. We followed a breeding programme described by Gaynor et al. (2017) with 21 years of a conventional phenotypic selection for yield (Fig. 1). We started with a coalescent simulation of whole-genome sequences for 21 chromosome pairs and extracted random 600 biallelic single-nucleotide polymorphisms (SNP) as markers per chromosome, and randomly assigned 100 SNP as QTL per chromosome. We assumed that the 2100 QTL had an additive effect on yield and sampled their effects from a normal distribution. We coded genotypes as 0 for reference (ancestral) homozygote, 1 for heterozygote and 2 for alternative (derived) homozygote. From the simulated whole-genome sequences, we created 70 inbred lines. The additive genetic variance between these inbred lines was set to 0.1. We crossed the inbred lines to generate 100 biparental populations. Each population had 100 F_1_ that had their genome doubled and planted in headrows (altogether 10,000). In the headrows, we visually evaluated the lines (trait heritability of 0.1) and advanced the best 500 into a preliminary yield trial. In the preliminary yield trial, we evaluated the lines in an unreplicated trial (trait heritability of 0.2) and advanced the best 50 into an advanced yield trial. In the advanced yield trial, we evaluated the lines in a small multi-location replicated trial (trait heritability of 0.5) and advanced the best 10 into an elite yield trial. In the elite yield trial, we evaluated the lines for two consecutive years in a large multi-location replicated trial (trait heritability of 0.67) and released one variety. We used the best lines from the advanced and elite yield trials as parents to start a new breeding cycle. During the breeding programme simulation process, we generated fully inbred individuals (except in the F_1_) and, as a consequence, we assumed only additive genetic variation because dominance variance is not visible in inbred individuals, while epistasis variance is generally small (e.g., Hem et al. 2021; Gonzalez-Dieguez et al. 2021).

We have saved phenotype and marker genotype data throughout the simulation to generate a training population for genomic modelling. For simplicity, we did not use the genomic data in simulation of selection but only saved it for a retrospective statistical analysis of temporal and genomic trends of genetic variance. To this end, we have constructed a training population that spanned the last 6 years of the simulation, from years 16 to 21. This training population covered 3070 lines with preliminary, advanced and elite yield trial phenotypes (altogether 3420 phenotypes) and corresponding genotypes at 10,500 markers.

### Temporal and genomic analysis of genetic variation

Here we describe a theoretical approach to temporal and genomic analysis of genetic variation, assuming we know the QTL genotypes and their effects. In the following sub-sections, we present a framework for temporal and genomic analysis of genetic variation that closely matches this theoretical approach; however, it uses observed phenotypes and marker genotypes to analyse the genetic variation. The theoretical approach consists of four steps. First, we define whole-genome genetic values from QTL genotypes and their effects, and then, we partition individuals and their genetic values by time to calculate genetic variances over these time partitions for temporal analysis. Finally, we partition whole-genome genetic values into chromosome and locus genetic values to calculate genetic variances and covariances over these genomic partitions for genomic analysis. This calculation involves three ‘layers’ of variances: (a) total (whole-genome) genetic variance, (b) chromosome variances alongside linkage-disequilibrium covariances between chromosomes and (c) locus genic variances alongside locus linkage-disequilibrium covariances within chromosomes and locus linkage-disequilibrium covariances between chromosomes. Fourth, we combine temporal and genomic analyses.

First, let ***Q*** be *n*_*i*_ × *n*_*q*_ matrix of QTL genotypes for *n*_*i*_ individuals at *n*_*q*_ QTL, with ***Q***[*i*, *l*] denoting QTL genotype of individual *i* at locus *l*. Also, let ***α*** be *n*_*q*_ × 1 vector of QTL additive effects, with ***α***[*l*] denoting QTL additive effect at locus *l*. Whole-genome genetic values of *n*_*i*_ individuals are a linear combination of QTL genotypes and their effects, ***a*** = ***Qα***, with ***a***[*i*] denoting genetic value of individual *i* and ***a***[*i* : *j*] denoting genetic values of a set of individuals spanning from the *i*th individual to the *j*th individual, inclusive, in the ***a***. Variance of these genetic values is genetic variance, $Var\left({{{{{\boldsymbol{a}}}}}}\right)=1/n\mathop{\sum }\nolimits_{i = 1}^{n}{\left({a}_{i}-1/n\mathop{\sum }\nolimits_{i = 1}^{n}{a}_{i}\right)}^{2}$. Note that this variance pertains to all *n*_*i*_ individuals and might not be an informative measure if these individuals span multiple stages and years of a breeding programme. In fact, directional selection or population structure will likely inflate this variance measure and mislead breeders in overestimating the amount of genetic variance. This is why we need temporal analysis of genetic variance.

Second, for the temporal analysis of genetic variance we partition the vector of genetic values by time and calculate variance for each time partition (Sorensen et al. 2001). For example, assume that individuals and their genetic values are ordered by time and that we partition them into time groups as $\left\{{{{{{\boldsymbol{a}}}}}}[1:k]\right.$, ***a***[(*k* + 1): *l*], ***a***[(*l* + 1): *m*], $\left.\ldots \right\}$. Then the temporal analysis of genetic variance is obtained by calculating variance for each time partition: ${\sigma }_{{a}_{1}}^{2}=Var\left({{{{{\boldsymbol{a}}}}}}[1:k]\right)$, ${\sigma }_{{a}_{2}}^{2}=Var\left({{{{{\boldsymbol{a}}}}}}[(k+1):l]\right)$, ${\sigma }_{{a}_{3}}^{2}=Var\left({{{{{\boldsymbol{a}}}}}}[(l+1):m]\right)$, ….

**Fig. 2:**
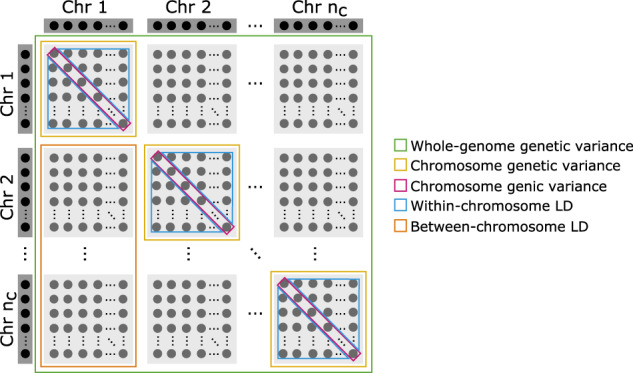
Genomic variance partitioning. Illustrative scheme of genomic partitioning of whole-genome genetic variance by chromosomes and loci into genic, and within- and between-chromosome linkage-disequilibrium (LD) components.

Third, for genomic analysis of genetic variance we initially partition whole-genome genetic values ***a*** into an *n*_*i*_ × *n*_*c*_ matrix of *n*_*c*_ chromosome genetic values ***A***_*c*_ such that the sum over chromosome genetic values gives whole-genome genetic values ${{{{{\boldsymbol{a}}}}}}=\mathop{\sum }\nolimits_{c = 1}^{{n}_{c}}{{{{{{\boldsymbol{A}}}}}}}_{c}[:,c]$. We obtain these chromosome genetic values by summing locus genetic values ***A***_*q*_ on each chromosome, ***A***_*c*_[*i*, *c*] = ∑_*l*_***Q***[*i*, *l*]***α***[*l*] for *l* running over ${n}_{{l}_{c}}$ QTL on a chromosome *c*. Note that ${{{{{\boldsymbol{a}}}}}}=\mathop{\sum }\nolimits_{q = 1}^{{n}_{q}}{{{{{{\boldsymbol{A}}}}}}}_{q}[:,q]=\mathop{\sum }\nolimits_{c = 1}^{{n}_{c}}{\sum }_{l}{{{{{{\boldsymbol{A}}}}}}}_{q}[:,l]$ for *l* running over ${n}_{{l}_{c}}$ QTL on a chromosome *c*. To calculate genetic variances over these genomic partitions we will use the variance sum rule *Var*(*x* + *y*) = *Var*(*x*) + *Var*(*y*) + 2*Cov*(*x*, *y*) and the variance product rule *Var*(*xa*) = *Var*(*x*)*a*^2^. Partitioning of the genetic variance ${\sigma }_{a}^{2}$ by chromosomes gives the sum of *n*_*c*_ chromosome variances $({\sigma }_{a,c}^{2})$ and ${n}_{c}\times \left({n}_{c}-1\right)$ covariances between chromosomes $\left({\sigma }_{(a,c^{\prime} )(a,c)}\right)$:

$$\begin{array}{ll}Var\left({{{{{\boldsymbol{a}}}}}}\right)={\sigma }_{a}^{2}=Var\left(\mathop{\sum }\limits_{c}^{{n}_{c}}{{{{{{\boldsymbol{A}}}}}}}_{c}[:,c]\right)={\sigma }_{a,1}^{2}+{\sigma }_{a,2}^{2}+\cdots +{\sigma }_{a,{n}_{c}}^{2}+\\ 2\left({\sigma }_{(a,2)(a,1)}+\cdots +{\sigma }_{(a,{n}_{c})(a,{n}_{c}-1)}\right),\end{array}$$

with covariances between chromosomes being between-chromosome linkage-disequilibrium covariances (Fig. 2). Partitioning of a chromosome genetic variance ${\sigma }_{a,c}^{2}$ by loci gives the sum of ${n}_{{l}_{c}}$ locus variances $({\sigma }_{a,c,l}^{2})$ and ${n}_{l}\times \left({n}_{l}-1\right)$ covariances between loci $\left({\sigma }_{(a,c,l^{\prime} )(a,c,l)}\right)$:

$${\sigma }_{a,c}^{2}={\sigma }_{a,c,1}^{2}+{\sigma }_{a,c,2}^{2}+\cdots +{\sigma }_{a,c,{n}_{{l}_{c}}}^{2}+2\left({\sigma }_{(a,c,2)(a,c,1)}+\cdots +{\sigma }_{(a,c,{n}_{{l}_{c}})(a,c,{n}_{{l}_{c}}-1)}\right),$$

with locus variances being genic variances and covariances between loci being within-chromosome linkage-disequilibrium covariances (Fig. 2) (Lynch and Walsh 1998; Walsh and Lynch 2018; Bulmer 1971). Locus genic variance is a function of variance in locus genotypes and their allele substitution effects (Falconer and Mackay 1996; Gianola et al. 2009) (using variance product rule):

$${\sigma }_{a,c,l}^{2}=Var\left({{{{{{\boldsymbol{A}}}}}}}_{q}[:,l]\right)=Var\left({{{{{\boldsymbol{Q}}}}}}[:,l]{{{{{\boldsymbol{\alpha }}}}}}[l]\right)={{{{{\boldsymbol{\alpha }}}}}}{[l]}^{T}Var\left({{{{{\boldsymbol{Q}}}}}}[:,l]\right){{{{{\boldsymbol{\alpha }}}}}}[l],$$

where we emphasise that we do not use the common Hardy–Weinberg assumption of $Var\left({{{{{\boldsymbol{Q}}}}}}[:,l]\right)=2{p}_{l}(1-{p}_{l})$ with *p*_*l*_ being allele frequency. Instead, we advocate to calculate empirical variance in observed locus genotypes, $Var\left({{{{{\boldsymbol{Q}}}}}}[:,l]\right)$. We will return to this point in the discussion. Note that genetic variance at a single-locus is the same as the genic variance. Locus linkage-disequilibrium covariance (=linkage-disequilibrium between locus genetic values) is a function of covariance between genotypes at two loci (=linkage-disequilibrium between locus genotypes) and their allele substitution effects:

$${\sigma }_{(a,c,l^{\prime} )(a,c,l)}={{{{{\boldsymbol{\alpha }}}}}}{[l^{\prime} ]}^{T}Cov\left({{{{{\boldsymbol{Q}}}}}}[:,l^{\prime} ],{{{{{\boldsymbol{Q}}}}}}[:,l]\right){{{{{\boldsymbol{\alpha }}}}}}[l].$$

We can now partition the whole-genome genetic variance over chromosomes and loci as a sum of locus genic variances, within-chromosome linkage-disequilibrium covariances and between-chromosome linkage-disequilibrium covariances (Fig. 2):

1 $$\begin{array}{lll}{\sigma }_{a}^{2}=&\mathop{\sum }\limits_{c=1}^{{n}_{c}}\mathop{\sum }\limits_{l=1}^{{n}_{{l}_{c}}}{\sigma }_{a,c,l}^{2}+&(=\,{{\mbox{genic variance}}}\,)\\ &2\mathop{\sum }\limits_{c=1}^{{n}_{c}}\mathop{\sum }\limits_{l=1}^{{n}_{{l}_{c}}-1}\mathop{\sum }\limits_{l^{\prime} =l+1}^{{n}_{{l}_{c}}}{\sigma }_{(a,c,l^{\prime} )(a,c,l)}+&(=\,{{\mbox{within-chromosome linkage-disequilibrium}}}\,)\\ &2\mathop{\sum }\limits_{c=1}^{{n}_{c}-1}\mathop{\sum }\limits_{c^{\prime} =c+1}^{{n}_{c}}\mathop{\sum }\limits_{l=1}^{{n}_{{l}_{c}}}\mathop{\sum }\limits_{l^{\prime} =l}^{{n}_{{l}_{c}}}{\sigma }_{(a,c^{\prime} ,l^{\prime} )(a,c,l)}.&(=\,{{\mbox{between-chromosome linkage-disequilibrium}}}\,)\end{array}$$

With *n*_*l*_ = 2100 QTL spread evenly over *n*_*c*_ = 21 chromosomes, the total number of locus combinations is *n*_*l*_ × *n*_*l*_ = 4,410,000 and the total number of chromosome combinations is *n*_*c*_ × *n*_*c*_ = 441. The theoretical approach partitions genetic variance into *n*_*l*_ = 2100 locus genic variances (*n*_*c*_ = 21 chromosome genic variances), ${n}_{c}\times {n}_{{l}_{c}}\times \left({n}_{{l}_{c}}-1\right)=207,900$ locus within-chromosome linkage-disequilibrium covariances (*n*_*c*_ = 21 chromosome within-chromosome linkage-disequilibrium covariances), and ${n}_{l}\times {n}_{l}-{n}_{c}\times {n}_{{l}_{c}}\times {n}_{{l}_{c}}=4,197,900$ locus between-chromosome linkage-disequilibrium covariances (*n*_*c*_ × *n*_*c*_ − *n*_*c*_ = 420 chromosome between-chromosome linkage-disequilibrium covariances). In this study we work only with the genome and chromosome level partitioning of genetic variance. For a genome region level partitioning, see Burch et al. (2021). We emphasise these numbers because we often hear colleagues saying that there is no or limited between-chromosome linkage-disequilibrium (due to the lack of physical linkage). However, selection and other population processes can generate non-zero within- and between-chromosome linkage-disequilibrium covariance (Lynch and Walsh 1998; Walsh and Lynch 2018; Rawlik et al. 2020; Bulmer, 1971). Even if the between-chromosome linkage-disequilibrium covariances are very small, there is a very large number of them and they can collectively have a sizeable effect on genetic variance as we show in results. It is important to emphasise the distinction between linkage-disequilibrium between locus genotypes $(Cov\left({{{{{\boldsymbol{Q}}}}}}[:,l^{\prime} ],{{{{{\boldsymbol{Q}}}}}}[:,l]\right))$ and linkage-disequilibrium between locus genetic values $({{{{{\boldsymbol{\alpha }}}}}}{[l^{\prime} ]}^{T}Cov({{{{{\boldsymbol{Q}}}}}}[:,l^{\prime} ],{{{{{\boldsymbol{Q}}}}}}[:,l]){{{{{\boldsymbol{\alpha }}}}}}[l])$. When looking at a whole-genome level, to obtain a non-zero linkage-disequilibrium covariance contribution to genetic variance, we require a non-zero linkage-disequilibrium between locus genotypes and a population process that couples this linkage-disequilibrium between locus genotypes with QTL effects. Selection or assortative mating are two such population processes because they are driven by QTL effects, while drift is not (Lynch and Walsh 1998; Walsh and Lynch 2018; Rawlik et al. 2019, 2020; Bulmer 1971).

Fourth, for the joint temporal and genomic analysis, we perform genomic partitioning and variance calculations for individuals and their genetic values partitioned by time.

### Statistical analysis of observed data

In the previous sub-section, we assumed we know the QTL and their effects. However, in reality, we observe phenotypes and marker genotypes and make inferences based on this information. To this end, we fitted the marker-based model (Meuwissen et al. 2001; Whittaker et al. 2000; de los Campos et al. 2013):

2 $$\begin{array}{l}{{{{{\boldsymbol{y}}}}}}={{{{{\boldsymbol{Xb}}}}}}+{{{{{\boldsymbol{ZWm}}}}}}+{{{{{\boldsymbol{e}}}}}},\\ {{{{{\boldsymbol{m}}}}}} \sim {{{{{\mathcal{N}}}}}}({{{{{\boldsymbol{0}}}}}},{{{{{{\boldsymbol{I}}}}}}}_{{n}_{m}}{\sigma }_{m}^{2}),\ \,{{\mbox{and}}}\,\ {{{{{\boldsymbol{e}}}}}} \sim {{{{{\mathcal{N}}}}}}({{{{{\boldsymbol{0}}}}}},{{{{{{\boldsymbol{I}}}}}}}_{{n}_{y}}{\sigma }_{e}^{2}),\end{array}$$

where, ***y*** is an *n*_*y*_ × 1 vector of *n*_*y*_ phenotypic values, ***X*** is an *n*_*y*_ × *n*_*b*_ incidence matrix associated with the intercept and *n*_*b*_ − 1 year effects ***b***, ***Z*** is an *n*_*y*_ × *n*_*i*_ incidence matrix for *n*_*i*_ lines whose marker genotype data are in an *n*_*i*_ × *n*_*m*_ matrix ***W*** for *n*_*m*_ marker effects ***m***, and ***e*** is an *n*_*y*_ × 1 vector of *n*_*y*_ residuals. In this study *n*_*y*_ was 3420, *n*_*b*_ was 6, *n*_*i*_ was 3070 and *n*_*m*_ was 10,500. We assumed that marker effects are a priori uncorrelated and normally distributed with zero mean and variance component describing variation between marker effects ${\sigma }_{m}^{2}$ (Eq. (2)). We further assumed that residuals are uncorrelated and normally distributed with zero mean and residual variance ${\sigma }_{e}^{2}$ (Eq. (2)). We ignored that different yield trials had different amount of replication and therefore different error variance.

The model (Eq. (2)) has location parameters (means) ***b*** and ***m*** and dispersion parameters (variances) ${\sigma }_{m}^{2}$ and ${\sigma }_{e}^{2}$. We emphasise that ${\sigma }_{m}^{2}$ is variance between marker effects and note that the commonly used approximation for ‘genomic variance’ ${\sigma }_{m}^{2}\times 2\mathop{\sum }\nolimits_{m=1}^{{n}_{m}}{p}_{m}(1-{p}_{m})$ (VanRaden 2008; Hayes et al. 2009) is scaled variance between marker effects and not genetic variance (Gianola et al. 2009; de los Campos et al. 2015; Rawlik et al. 2020). The scaling factor is the sum of expected variances for marker genotypes assuming Hardy–Weinberg equilibrium. Comparison of this approximation with Eq. (1) shows that the approximation ignores linkage-disequilibrium and non-Hardy–Weinberg components of genetic variance as well as uses variance between marker effects instead of QTL effects. However, linkage-disequilibrium affects the estimate of variance between marker effects. At any rate, this estimate of genetic variance is not amenable for our aim of doing temporal or genomic analyses. We view variance between marker effects simply as a statistical/model parameter that facilitates model fitting to observed data. We describe the model fitting and estimation of variances in the next sub-section.

### Statistical and computational approaches

We used the empirical and full Bayesian approach to estimate the model’s parameters (Eq. (2)) with marker genotypes or their leading principal components. Given the variances ${\sigma }_{m}^{2}$ and ${\sigma }_{e}^{2}$, we can estimate fixed effects ***b*** and marker effects ***m*** by solving Henderson’s mixed model equations:

3 $$\left[\begin{array}{ll}{{{{{{\boldsymbol{X}}}}}}}^{T}{{{{{\boldsymbol{X}}}}}}&{{{{{{\boldsymbol{X}}}}}}}^{T}{{{{{\boldsymbol{ZW}}}}}}\\ {{{{{{\boldsymbol{W}}}}}}}^{T}{{{{{{\boldsymbol{Z}}}}}}}^{T}{{{{{\boldsymbol{X}}}}}}&{{{{{{\boldsymbol{Z}}}}}}}^{T}{{{{{{\boldsymbol{W}}}}}}}^{T}{{{{{\boldsymbol{W}}}}}}{{{{{\boldsymbol{Z}}}}}}+{{{{{\boldsymbol{I}}}}}}{\sigma }_{e}^{2}{\sigma }_{m}^{-2}\end{array}\right]\left[\begin{array}{l}\hat{{{{{{\boldsymbol{b}}}}}}}\\ \hat{{{{{{\boldsymbol{m}}}}}}}\end{array}\right]=\left[\begin{array}{l}{{{{{{\boldsymbol{X}}}}}}}^{T}{{{{{\boldsymbol{y}}}}}}\\ {{{{{{\boldsymbol{Z}}}}}}}^{T}{{{{{{\boldsymbol{W}}}}}}}^{T}{{{{{\boldsymbol{y}}}}}}\end{array}\right].$$

Specifically, the solution of Eq. (3) is the conditional expectation $(\hat{{{{{{\boldsymbol{b}}}}}}},\hat{{{{{{\boldsymbol{m}}}}}}})=E\left({{{{{\boldsymbol{b}}}}}},{{{{{\boldsymbol{m}}}}}}| {{{{{\boldsymbol{y}}}}}},{\sigma }_{m}^{2},{\sigma }_{e}^{2}\right)$. With these estimates we can obtain estimates of genetic values as $\hat{{{{{{\boldsymbol{a}}}}}}}={{{{{\boldsymbol{W}}}}}}\hat{{{{{{\boldsymbol{m}}}}}}}$. These estimates have some error and ignoring it in the framework will underestimate genetic variance. To see this, imagine we have very little phenotypic information such that marker estimates will effectively follow the prior Eq. (2). In that case, marker estimates will effectively all equal zero and any variance calculation will return a zero. As shown by Sorensen et al. (2001) and Lehermeier et al. (2017), we can account for this uncertainty by estimating genetic variances from posterior samples of genetic values or marker effects. For the model (Eq. (2) and (3)), we can obtain posterior samples from the multivariate normal distribution:

4 $${{{{{\mathcal{N}}}}}}\left(E\left({{{{{\boldsymbol{b}}}}}},{{{{{\boldsymbol{m}}}}}}| {{{{{\boldsymbol{y}}}}}},{\sigma }_{m}^{2},{\sigma }_{e}^{2}\right),Var\left({{{{{\boldsymbol{b}}}}}},{{{{{\boldsymbol{m}}}}}}| {{{{{\boldsymbol{y}}}}}},{\sigma }_{m}^{2},{\sigma }_{e}^{2}\right)\right),$$

where conditional variance $Var({{{{{\boldsymbol{b}}}}}},{{{{{\boldsymbol{m}}}}}}| {{{{{\boldsymbol{y}}}}}},{\sigma }_{m}^{2},{\sigma }_{e}^{2})$ can be obtained by solving the left-hand-side of the system of equations (Eq. (3)) (Sorensen and Gianola 2007).

Once we obtained samples of marker effects from Eq. (4), we have treated marker genotypes and marker effects respectively as if they were QTL genotypes and QTL effects and analysed temporal and genomic trends in genetic variance as described in the theoretical sub-section. Specifically, for each sample of marker effects, we have estimated genetic values and their variance for each group of individuals in the breeding programme (parents, F_1_ progeny, headrows, etc.) for each year for the temporal analysis and further partitioned along the genome for the genomic analysis. This procedure gave us posterior distribution for the genetic variance of each group, time and genome partition.

When variances are unknown, we can use the empirical Bayesian approach (Sorensen and Gianola 2007; Efron 1996) and estimate most likely variances given the data and use them to calculate conditional expectation and variance as well as draw samples from Eq. (4). Alternatively, we can use the full Bayesian approach by specifying prior distribution for all model parameters and obtain posterior distribution $p({{{{{\boldsymbol{b}}}}}},{{{{{\boldsymbol{m}}}}}},{\sigma }_{m}^{2},{\sigma }_{e}^{2}| {{{{{\boldsymbol{y}}}}}})\propto p({{{{{\boldsymbol{y}}}}}}| {{{{{\boldsymbol{b}}}}}},{{{{{\boldsymbol{m}}}}}},{\sigma }_{e}^{2})p({{{{{\boldsymbol{b}}}}}}| {\sigma }_{b}^{2})p({{{{{\boldsymbol{m}}}}}}| {\sigma }_{m}^{2})p({\sigma }_{b}^{2})p({\sigma }_{m}^{2})p({\sigma }_{e}^{2})$ (Sorensen and Gianola 2007).

We fitted the model (Eq. (2)) both with the full and the empirical Bayesian approach. We first used MCMC for the full Bayesian approach and used one chain with 100,000 samples, 10,000 burn-in and saved every 100th sample to obtain 900 samples of all model parameters. For the empirical Bayesian approach, we also obtained 900 samples but used posterior mean for the marker effect and residual variances estimated from the full Bayesian approach when sampling from Eq. (4).

Since genomic analyses can be time-consuming, we have also investigated the use of approximation for marker genotypes with their leading principal components. We changed the model (Eq. (2)) into:

5 $$\begin{array}{l}{{{{{\boldsymbol{y}}}}}}={{{{{\boldsymbol{Xb}}}}}}+{{{{{\boldsymbol{ZTs}}}}}}+{{{{{\boldsymbol{e}}}}}},\\ {{{{{\boldsymbol{s}}}}}} \sim N({{{{{\boldsymbol{0}}}}}},{{{{{{\boldsymbol{I}}}}}}}_{{n}_{p}}{\sigma }_{s}^{2}),\ \,{{\mbox{and}}}\,\ {{{{{\boldsymbol{e}}}}}} \sim N({{{{{\boldsymbol{0}}}}}},{{{{{{\boldsymbol{I}}}}}}}_{{n}_{y}}{\sigma }_{e}^{2}),\end{array}$$

where ***T*** is an *n*_*i*_ × *n*_*p*_ score matrix obtained from a truncated SVD of genotypes with the *n*_*p*_ leading principal components such that ${{{{{{\boldsymbol{T}}}}}}}_{({n}_{i}\times {n}_{p})}={{{{{{\boldsymbol{U}}}}}}}_{({n}_{i}\times {n}_{p})}{{{{{{\boldsymbol{S}}}}}}}_{({n}_{p}\times {n}_{p})}={{{{{{\boldsymbol{U}}}}}}}_{({n}_{i}\times {n}_{p})}{{{{{{\boldsymbol{S}}}}}}}_{({n}_{p}\times {n}_{p})}{{{{{{\boldsymbol{V}}}}}}}_{({n}_{m}\times {n}_{p})}^{T}{{{{{{\boldsymbol{V}}}}}}}_{({n}_{m}\times {n}_{p})}={{{{{{\boldsymbol{W}}}}}}}_{({n}_{i}\times {n}_{m})}{{{{{{\boldsymbol{V}}}}}}}_{({n}_{m}\times {n}_{p})}$, ***s*** is an *n*_*p*_ × 1 vector of *n*_*p*_ principal component effects and ${\sigma }_{s}^{2}$ is variance between principal component effects (Tusell et al. 2013; Ødegård et al. 2018; Hastie and Tibshirani 2004). The ***S*** matrix is a diagonal matrix with *n*_*p*_ singular values (square root of non-zero eigenvalues of ***W***^*T*^***W*** and ***WW***^*T*^), the columns of ***U*** are left singular vectors, and the columns of ***V*** are right singular values. This model is structurally the same as the model (Eq. (2)) and we fitted it in the same way. We approximated marker effect samples by ***m***^*i*^ = ***Vs***^*i*^, where ***s***^*i*^ is the *i*th sample of principal component effects. Once we approximated marker effect samples we used the same approach as described above. We investigated different number of principal components (10, 50, 100, 500, 1000, 2000 and 3420). In our simulation these numbers of principal components respectively explained 14%, 38%, 52%, 84%, 94%, 99% and 100% of marker genotype variation in the first replicate.

### Sensitivity to genetic architecture

To test our framework’s sensitivity to different genetic architectures, we have done additional simulations by varying the number of QTL and by adding genotype-by-environment interactions. Namely, the framework will depend on the ability of the fitted statistical model to capture the genetic complexity of the analysed trait. We have simulated an additive trait with either 10, 100 or 1000 QTL per chromosome, respectively with 210, 2100 or 21000 QTL per genome. In addition, we have added variation in QTL effects across years for genotype-by-environment interactions across years, assuming that years represent different environments. The amount of this additional phenotype variance due to genotype-by-year interactions was set to 0.2. In total, this gave us six scenarios (three for the number of QTL and two for absence/presence of genotype-by-year interactions). In each of the scenarios, we used the standard model (Eq. (2)) that is ignorant about the number of QTL or the presence of genotype-by-year interactions.

### Summarising the results

We compared how obtained posterior distributions for genetic variances and their components match the true values from simulation. We also calculated the continuous ranked probability score (CRPS) (Gneiting and Raftery 2007) to compare whole posterior distributions to true values to assess both accuracy and precision and with this account for the uncertainty of estimation. For an intuitive description of CRPS, see Selle et al. (2019). Finally, we also calculated the concordance correlation coefficient (CCC) (Lin 1989) to additionally assess agreement between the true and estimated values of genetic and genic variance in some analyses. We used CCC because it has two clear components—the Pearson correlation coefficient indicating precision (closeness to the best-fit line between the true and estimated values) and the bias correction factor indicating accuracy (closeness to the equality line between the true and estimated values).

### Software implementation

We have simulated the wheat breeding programme using the R package AlphaSimR (Gaynor et al. 2021). We have fitted the model with the AlphaBayes software (source code at https://github.com/AlphaGenes/alphabayes) (Gorjanc and Hickey 2019). We used R (R Core Team 2019) for post-processing the AlphaBayes marker effect samples and further analyses. To calculate the CRPS (Jordan et al. 2019), we used the scoringRules R package. To calculate the CCC (Lin 1989), we used the DescTools R package (Signorell et al. 2021).

## Results

Overall, the results show that estimates from the data following the framework were in concordance with the true values for temporal and genomic analysis, provided that the fitted model is capturing the genetic complexity of a trait. We separate the result section into four areas to facilitate presentation: (1) temporal analysis, (2) genomic analysis, (3) computational analysis and (4) sensitivity to genetic architecture.

### Temporal analysis

**Fig. 3:**
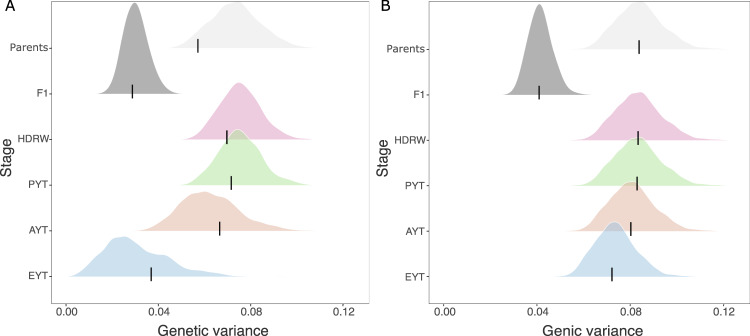
Breeding analysis. Genetic (**A**) and genic (**B**) variance estimated with the full Bayesian approach for parents in year 16, F_1_ progeny (F1) in year 17, headrows (HDRW) in year 18, preliminary yield trial (PYT) in year 19, advanced yield trial (AYT) in year 20 and elite yield trial (EYT) in year 21; black lines denote the true values and densities depict posterior distributions.

The genetic and genic variance changed through the breeding cycle. We show this in Fig. 3 with the true and estimated genetic and genic variances for different stages of one breeding cycle (see breeding scheme on the left in Fig. 1). As expected, genetic variation in F_1_ progeny across multiple crosses was lower than in the parents as this variance indicates variance in parent averages between crosses. When we generated doubled haploids for these full-sib families (HDRW stage), genetic variation was regenerated to the level in parents due to recombination and complete inbreeding. Genetic variation gradually reduced through the breeding cycle due to the repeated selection from headrows to elite yield trial. This change was particularly evident for genetic variance but less for genic variance. Also, the genetic variance was consistently smaller than the genic variance. The estimated genetic and genic variance matched the true values well across all breeding stages. There was more uncertainty in the estimates of genetic variance in the elite yield trial than in other stages.

**Fig. 4:**
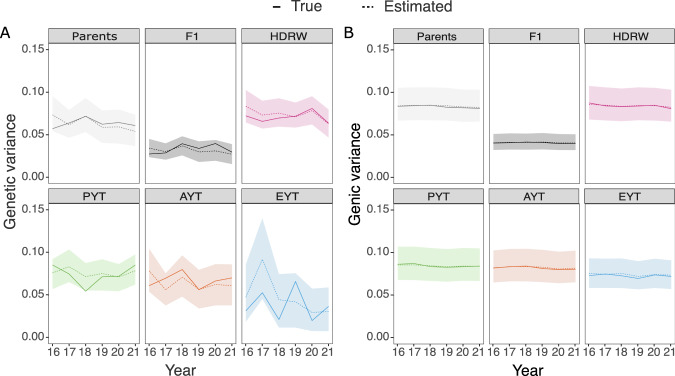
Temporal analysis. Temporal trends in genetic (**A**) and genic (**B**) variance estimated with the full Bayesian approach for parents, F_1_ progeny (F1), headrows (HDRW), preliminary yield trial (PYT), advanced yield trial (AYT) and elite yield trial (EYT); solid lines denote the true value, dashed lines denote posterior means and polygons depict 95% posterior quantiles.

Genetic variation decreased over the years and genetic variance was consistently smaller and more variable than genic variance across years. We show this in Fig. 4 with the true and estimated temporal trends of genetic and genic variances for different breeding stages (see temporal scheme on the right in Fig. 1). Variances between the breeding stages differed as mentioned before, but in Fig. 4 we also see a consistent decrease over the years, which was variable for genetic variance but not for genic variance. Furthermore, this variability increased from early to late breeding stages as fewer and fewer individuals were in a stage. Thus, the genetic and genic variance estimates have matched the true values very well across all breeding stages and years.

### Genomic analysis

**Fig. 5:**
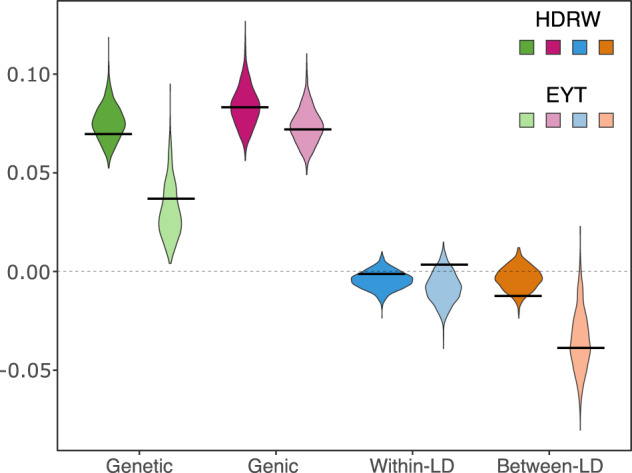
Genomic analysis. Whole-genome genetic and genic variances, and within- and between-chromosome linkage-disequilibrium (LD) covariances with the full Bayesian approach for headrows (HDRW, year 18) and elite yield trial (EYT, year 21); genetic variance is the sum of genic variance, within- and between-chromosome LD (see Fig. 2); black lines denote true values and violins depict posterior distributions.

The genomic analysis enabled accurate partitioning of whole-genome genetic variance into whole-genome genic variance and whole-genome linkage-disequilibrium covariances. We show this in Fig. 5 with true and estimated variances and covariances for headrows and elite yield trial from one breeding cycle. Figure 5 shows, as previously described, differences in genetic and genic variances as well as a substantial change in the between-chromosome linkage-disequilibrium covariance, which was the main driver of change in genetic variance between headrows and the elite yield trial. Specifically, genetic variance decreased from 0.0754 in headrows in year 18 to 0.0307 in the elite yield trial in year 21, with a change of 0.0447 (59% reduction). This overall change was due to 0.01 change in genic variance (22% of the initial genetic variance), 0.0036 change in within-chromosome linkage-disequilibrium covariance (8% of the initial genetic variance) and 0.0311 change in between-chromosome linkage-disequilibrium covariance (70% of the initial genetic variance). The estimates matched the true values well. Supplementary Fig. S1 shows temporal trends for within-chromosome and between-chromosome linkage-disequilibrium. Between-chromosome linkage-disequilibrium varied more and decreased over time.

The genomic analysis also enabled accurate partitioning of whole-genome genetic variance by chromosomes. We show this in the Supplementary material with a series of Supplementary Tables S1–S4 and Supplementary Fig. S2. These supplements show genetic variance and its components (genic variance, within-chromosome linkage-disequilibrium covariance and between-chromosome linkage-disequilibrium covariance) by 21 chromosomes and how these values add up to the whole-genome variance. Specifically, the genetic variance of a quantitative trait is composed of (i) variation at the QTL genotypes (Supplementary Table S1) and (ii) variation of QTL effects, which combined with variation at the QTL genotypes gives the genetic variance of a quantitative trait (Supplementary Table S2). However, in reality we do not know QTL, we only know SNP markers, so we can only calculate (iii) variation at the genotypes of SNP markers (Supplementary Table S3) and (iv) estimate SNP marker effects, which combined with variation at the SNP marker genotypes gives an estimate of the genetic variance of a quantitative trait (Supplementary Table S4). Therefore, we are showing partitioning of genetic variance for QTL genotypes (Supplementary Table S1), marker genotypes (Supplementary Table S3), true genetic values (Supplementary Table S2) and estimated genetic values (Supplementary Table S4). The variance at QTL genotypes (Supplementary Table S1) and SNP marker genotypes (Supplementary Table S3) will likely differ because they are respectively a function of the number of QTL and SNP markers and their respective variation. Supplementary Table S1 reports genetic variance of QTL genotypes across all chromosomes of 1213.1 (this is based on 2100 QTL), while Supplementary Table S3 reports genetic variance of SNP marker genotypes across all chromosomes of 6452.6 (this is based on 10,500 SNP markers). Our aim is that the true genetic variance for the quantitative trait (Supplementary Table S2) and its estimate from SNP markers (Supplementary Table S4) will be similar. Supplementary Table S2 reports genetic variance for the quantitative trait of 0.082 (the true value), while Supplementary Table S4 reports estimated genetic variance from our framework of 0.079. The true and estimated values match well and the same holds for individual chromosomes, but there is larger variation, which is expected because there is less information per chromosome.

Supplementary Fig. S2 compares the true and estimated genetic variances directly. The Supplementary material, along with Supplementary Tables S1–S4, aims to demonstrate how we estimate variation in true genetic values, which is driven by unknown QTL and unknown QTL effects, by using marker genotypes and estimated marker effects. We make five observations. First, the analysis of QTL genotypes showed that whole-genome and chromosome genetic variance in unselected headrows is largely driven by genic variance, but there are some chromosomes with a substantial within-chromosome or between-chromosome linkage-disequilibrium covariance (Supplementary Table S1). Second, the magnitude of linkage-disequilibrium covariances increased in the elite yield trial, which reduced the whole-genome genetic variance; however, between-chromosome linkage-disequilibrium was larger than within-chromosome linkage-disequilibrium (Supplementary Table S1). Third, the analysis of marker genotypes followed the same trends, but the values were sustainability larger due to the larger number of markers than QTL (Supplementary Table S3). Fourth, the analysis of true genetic values resulted in much smaller values for variances than the analysis of QTL genotypes because most QTL have small effects, but the relative magnitude of variation and their partitioning were similar (Supplementary Table S2). Fifth, the analysis of variance of estimated genetic values followed the analysis of variance of true genetic values closely—most deviations were observed for the elite yield trial, but all posterior distributions encompassed the true value (Supplementary Table S4). This analysis pertains to one single dataset to show that estimates are reasonable for a specific dataset.

### Computational analysis

**Fig. 6:**
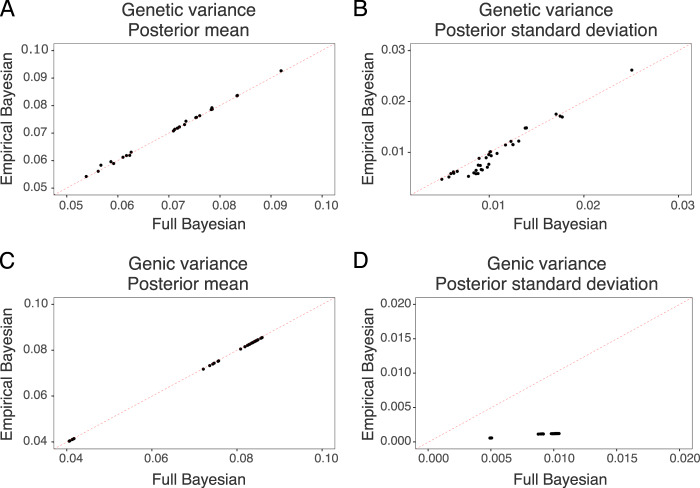
Statistical analysis. The empirical Bayesian approach versus the full Bayesian approach for posterior mean of genetic variance (**A**), posterior mean of genic variance (**B**), posterior standard deviation of genetic variance (**C**) and posterior standard deviation of genic variance (**D**); equal value is represented by the dashed red line.

Full and empirical Bayesian approaches had similar posterior mean estimates of variances, but the empirical Bayesian approach had smaller posterior standard deviation. We show this in Fig. 6 with a comparison of posterior means and posterior standard deviations for genetic and genic variance between the two approaches. The posterior means matched well for both types of variances. However, the posterior standard deviation was smaller with the empirical Bayesian approach, particularly for the genic variance. Comparison with the true values, however, showed good concordance with the empirical Bayesian posterior means (Supplementary Figs. S3 and S4).

Additional evaluation with multiple replicates showed that the full and empirical Bayesian results were consistently estimated for genetic and genic variance. We show this in Table 1 with CRPS of genetic and genic variances for full and empirical Bayesian approaches by breeding stage. Note that CRPS is negatively oriented—lower values indicate better estimates compared to the true value in terms of accuracy and precision. CRPS for genetic variance matched closely between the full and empirical Bayesian approaches. On the other hand, they differ more for genic variance, with better (lower) values for the full Bayesian approach, albeit there was considerable variability across years and replicates. Moreover, CRPS was larger (worse) for genic variance than for genetic variance.

**Table 1:** Continuous ranked probability score (CRPS × 1000 – lower is better: mean ± standard deviation over 6 years and ten replicates) for genetic and genic variance estimated by the full Bayesian and the empirical Bayesian for parents, F_1_ progeny, headrows (HDRW), preliminary yield trial (PYT), advanced yield trial (AYT) and elite yield trial (EYT).

	Genetic		Genic	
Stage	Full	Empirical	Full	Empirical
Parents	59 ± 40	60 ± 41	300 ± 93	351 ± 97
F_1_	42 ± 39	42 ± 40	40 ± 44	48 ± 52
HDRW	45 ± 32	46 ± 37	297 ± 94	348 ± 99
PYT	63 ± 57	64 ± 64	296 ± 94	348 ± 98
AYT	66 ± 63	66 ± 64	294 ± 92	344 ± 97
EYT	79 ± 45	80 ± 46	70 ± 75	84 ± 90

**Fig. 7:**
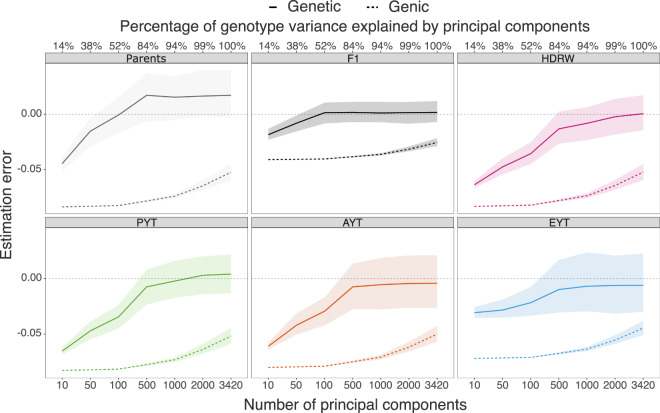
Approximation error with dimension reduction. Estimation error in genetic and genic variances as a function of the number of principal components in parents in year 16, F_1_ progeny (F1) in year 17, headrows (HDRW) in year 18, preliminary yield trial (PYT) in year 19, advanced yield trial (AYT) in year 20 and elite yield trial (EYT) in year 21; horizontal dashed line represents no estimation error.

When we used a sufficient number of principal components, approximation with leading principal components accurately estimated genetic variance, but this was never the case for genic variance. We show this in Fig. 7 with estimation error, defined as the difference between the true and estimated value, for genetic and genic variance as a function of the number of leading principal components. The estimation error decreased as we increased the number of leading principal components. It decreased quickly for the genetic variance—there was no error once we captured about 80% of the variation in marker genotypes. In our simulated dataset from the first replicate, we achieved this with 500 leading principal components. On the other hand, the estimation error decreased slowly for the genic variance, and we never recovered the true estimate, even if we used all the principal components. The estimation error was smallest in the F_1_ progeny, followed by the elite yield trial, while the largest estimation error was in the parents.

### Sensitivity to genetic architecture

Our framework relies on a statistical model that can capture the genetic complexity of an analysed trait. We have tested the effect of using a sub-optimal statistical model by varying the number of QTL and by adding genotype-by-year interaction in our simulation, without accounting for these complexities in the statistical model. Results in Supplementary Figs. S5 and S6 and Supplementary Table S5 show that variance estimates are very sensitive to genotype-by-year interaction and less to the number of QTL.

For scenarios without genotype-by-year interaction, the estimates of genetic and genic variance were very much in line with the true values and largely insensitive to the number of QTL (Supplementary Figs. S5 and S6 and Supplementary Table S5). Furthermore, concordance correlation and its two components (Pearson correlation and bias correction factor—Supplementary Table S5) showed good agreement between the true and estimated values for genetic and genic variances, with high precision and low bias.

For scenarios with genotype-by-year interaction, we can see substantial overestimation of genetic and genic variances (Supplementary Figs. S5 and S6). This overestimation decreased as the number of QTL increased, but even with 21,000 QTL, we still overestimated the variances by as much as 200%. Estimates of genic variance showed systematically consistent overestimation, while estimates of genetic variance were more variable. Concordance correlation and its two components (Pearson correlation and bias correction factor—Supplementary Table S5) showed low to moderate agreement between true and estimated values for genetic and genic variances. The Pearson correlation for genic variance was high, which indicates precise estimation. Although the true and estimated genic variances had a high linear relationship (high Pearson correlation), their estimates were biased (low bias correction factor) (Supplementary Table S5). For genetic estimates, we can see a moderate Pearson correlation and a moderate bias correction factor. Therefore, the addition of genotype-by-year interaction biased the genetic and genic variances estimates but decreased the precision for only genetic variances.

## Discussion

The results show that the framework for temporal and genomic analysis of genetic variation is flexible, accurate and enables assessing the sustainability of a breeding programme as well as population processes that change genetic variance. These results highlight four topics for discussion in line with the structure of results: (1) temporal analysis of genetic variance, (2) genomic analysis of genetic variance, (3) computational aspects and (4) assumptions of this study.

### Temporal analysis

This study will help breeders assess the amount of genetic variance in their programmes and better manage its utilisation for future genetic gains. Genetic variance (specifically its square root) is a key component of the breeder’s equation for predicting response to selection (Lush 1937; Falconer and Mackay 1996). While breeding programmes routinely estimate genetic variance for traits under selection, most estimates pertain to a group of individuals that is arguably not the most relevant for routine breeding (Piepho et al. 2008). Specifically, with the pedigree-based model, the estimate of genetic variance pertains to pedigree founders, which can be several generations removed from currently interesting individuals. Furthermore, pedigree founders often span multiple generations due to incomplete pedigrees and, as such, the corresponding estimate of genetic variance does not have a clearly defined time point. Estimates of genetic variance from genome-based models pertain to the group of individuals for which the allele frequencies were computed—usually for the genotyped individuals or base population, both of which again do not have a clearly defined time point. In addition, the ‘genomic variance’ is estimating genetic variance only under some conditions (Gianola et al. 2009; de los Campos et al. 2015; Rawlik et al. 2020; Schreck et al. 2019). Therefore, we propose an alternative framework for temporal and genomic analyses of genetic variation.

The framework builds on Sorensen et al. (2001), Lehermeier et al. (2017) and Allier et al. (2019) to enable a straightforward temporal analysis of a breeding programme. The framework uses all the available data spanning multiple years (generations) to estimate model parameters, which are used to infer genetic values and their variances. Such flexibility of using all data but producing estimates for any group of individuals is crucial to inform breeders how much genetic variance they have at hand and to react accordingly. Possible reactions to a temporal analysis by a breeder could be (i) keeping the current breeding programme as it is, (ii) implementing active management of genetic variance using techniques such as optimal contribution selection (e.g., Woolliams et al. 2015; Akdemir and Sanchez 2016; Gorjanc et al. 2018; Akdemir et al. 2019), (iii) germplasm exchange with other programmes or, in the extreme, (iv) introgressing landrace germplasm (e.g., Gorjanc et al. 2016). For example, temporal trends in genetic and genic variance enable straightforward trait-specific estimation of effective population size (Gorjanc et al. 2018). Using this approach in this study, we estimate the effective population size for the parents at 111. This estimate suggests that the simulated breeding programme is sustainable (Falconer and Mackay 1996; Lynch and Walsh 1998; Walsh and Lynch 2018; Hill 2016) as corroborated by small changes in genetic variance between years.

There are also other approaches to the temporal analysis of genetic variance. Tsuruta et al. (2004) used the random regression to model genetic values and their variance over the years, and Hidalgo et al. (2020) used sliding time intervals in the same fashion. Both methods have some drawbacks—random regression can be computationally demanding, while time intervals must be sufficiently large to obtain accurate estimates. These two approaches respectively enrich the model or slice the data to estimate genetic variances over time as model parameters, while our framework treats model variance parameters and genetic variances over time separately. We will return to these differences at the end of the discussion. Hidalgo et al. (2020) used sliding time intervals to investigate changes in genetic (co)variances for a breeding programme that recently implemented genomic selection. They observed rapid changes in genetic (co)variances with the implementation of genomic selection. Their results highlight a need for breeder’s reaction and further analysis. One such analysis should be related to which components of genetic variance changed due to genomic selection.

### Genomic analysis

The proposed framework can estimate the size and trends for genomic components of genetic variance. We have followed a standard quantitative genetics decomposition of genetic variance (Lynch and Walsh 1998; Walsh and Lynch 2018; Gianola et al. 2009; Bulmer 1971). This decomposition involves variance of genotypes and their allele substitution effects at every locus (genic variance) and covariance between genotypes and their allele substitution effects between pairs of loci. The covariance can be further partitioned into covariance between loci on one chromosome (within-chromosome linkage-disequilibrium covariance) and covariance between loci on different chromosomes (between-chromosome linkage-disequilibrium covariance). We showed this decomposition for the variance of QTL genotypes (Supplementary Table S1), marker genotypes (Supplementary Table S3), true genetic values (Supplementary Table S2) and estimated values (Supplementary Table S4), all at the whole-genome and chromosome level. These results confirmed the prediction of Bulmer (1971) that directional selection on total genetic values or downstream phenotypes induces negative linkage-disequilibrium between QTL and other loci around them and that this component can cause rapid and major changes in genetic variance (Lynch and Walsh 1998; Walsh and Lynch 2018). We note that this negative linkage-disequilibrium is a function of genotype combinations between loci as well as their allele substitution effects. Therefore, we have to distinguish two types of linkage-disequilibrium: (i) linkage-disequilibrium between locus genotypes $\left(Cov\left({{{{{\boldsymbol{Q}}}}}}[:,l^{\prime} ],{{{{{\boldsymbol{Q}}}}}}[:,l]\right)\right)$, which is a function of allele frequencies at loci and correlation between loci (this linkage-disequilibrium is trait agnostic); and (ii) linkage-disequilibrium between locus genetic values $\left({{{{{\boldsymbol{\alpha }}}}}}{[l^{\prime} ]}^{T}Cov\left({{{{{\boldsymbol{Q}}}}}}[:,l^{\prime} ],{{{{{\boldsymbol{Q}}}}}}[:,l]\right){{{{{\boldsymbol{\alpha }}}}}}[l^{\prime} ]\right)$, which is a function of allele frequencies at loci, correlation between loci and allele substitution effects of the loci. Only linkage-disequilibrium between locus genetic values contributes to the genetic variance of a trait. As mentioned in methods, linkage-disequilibrium between locus genetic values is induced by population processes that are driven by QTL effects. For example, selection on a trait will induce linkage-disequilibrium between locus genetic values because the trait is influenced by QTL effects. On the other hand, drift will induce linkage-disequilibrium between locus genotypes, but not linkage-disequilibrium between locus genetic values because drift is not driven by QTL effects. While drift can generate sporadic linkage-disequilibrium between locus genetic values, the sum of such terms will tend to zero for traits with a large number of QTL. To see this behaviour, consider linkage-disequilibrium between locus genetic values $\left({{{{{\boldsymbol{\alpha }}}}}}{[l^{\prime} ]}^{T}Cov\left({{{{{\boldsymbol{Q}}}}}}[:,l^{\prime} ],{{{{{\boldsymbol{Q}}}}}}[:,l]\right){{{{{\boldsymbol{\alpha }}}}}}[l]\right)$ and summing such terms over all pairs of loci. Even if there is non-zero linkage-disequilibrium between locus genotypes, the multiplication with seemingly randomly allocated QTL effects (since linkage-disequilibrium between genotypes is not driven by QTL effects) will drive the sum to zero. This ‘cancelling out’ will diminish when we analyse smaller genome regions or traits with a smaller number of QTL.

The importance of linkage-disequilibrium in estimating genetic variance with genomic data is growing (de los Campos et al. 2015; Lehermeier et al. 2017; Allier et al. 2019). Our study added to this literature with simulation and demonstrating temporal changes in linkage-disequilibrium under selection both within one breeding cycle (headrows to elite yield trial) (Fig. 3) and between breeding cycles over the years (Fig. 4). We observed more considerable changes within breeding cycles than between, which can be explained by strong repeated selection within cycles and recombination among parent genomes between cycles. Interestingly, we observed large between-chromosome linkage-disequilibrium covariance in comparison to within-chromosome (Fig. 5). This observation is at odds with physical linkage between loci within a chromosome and no physical linkage between loci on separate chromosomes. Our explanation is that there are more combinations between loci on separate chromosomes than within chromosomes. Furthermore, limited recombination constrains selection to induce large changes in linkage-disequilibrium within chromosomes compared to between chromosomes. To put this into perspective, in the analysed example, we observed a 59% change in genetic variance within a breeding cycle (headrows to elite yield trial). Of this change, 13% (22% relative) was due to the change in genic variance, 5% (8% relative) was due to the change in within-chromosome linkage-disequilibrium covariance and 41% (70% relative) was due to the change in between-chromosome linkage-disequilibrium covariance (Fig. 5). Even though we randomly placed QTL and randomly allocated QTL effects from one distribution (in the founding population before selection), the components of genetic variance varied considerably between chromosomes. These assumptions are likely too simple. Indeed, Allier et al. (2019) observed substantial variation between chromosomes in maize. These results indicate that linkage-disequilibrium is an important component of the genetic variance in line with the theoretical work of Bulmer (1971) and Mather and Jinks (2013).

We expected underestimation of genic variance in this breeding study but have not observed this. We have simulated a breeding programme with directional selection, which induces negative linkage-disequilibrium (Bulmer 1971) due to repulsion linkage of QTL (Mather and Jinks 2013). We expected that repulsion linkage would ‘hide’ variation in some genome regions (due to a lack of variation in haplotypes) and, therefore, underestimate genic variance. We likely did not observe underestimation because the effective population was reasonably large (111), the selection was not too strong or there was a sufficient number of markers. However, across multiple replicates, the CRPS was worse for genic than genetic variance, which indicates that estimating genic variance is more challenging than for genetic variance.

The proposed framework for genomic analysis of genetic variance will pave the way for analysing population processes that change the variance. While selection induces linkage-disequilibrium between loci, it also changes allele frequencies (Lynch and Walsh 1998; Walsh and Lynch 2018; Bulmer 1971; Gorjanc et al. 2015). Interestingly, we have not observed major changes in genic variance, suggesting that allele frequencies have not changed much. However, we must emphasise that we have reported genic variance for all loci, i.e., the sum of all locus-specific genic variances. This total genic variance is likely to change far less than genic variance at individual loci because at some loci allele frequency might change away from 0.5 (genic variance at these loci will decrease), but at other loci, allele frequency might change towards 0.5 (genic variance at these loci will increase) (Crow 2010). Therefore, when we sum locus-specific genic variances, the total genic variance will not change much. More research is needed to study trends in allele frequencies for old and recent mutations, their locus-specific genic variances, and total genic variance.

Another important population process is drift, which is always present in breeding programmes due to small effective population sizes. However, distinguishing between selection and drift in such populations is difficult (Lynch and Walsh 1998; Walsh and Lynch 2018; Gorjanc et al. 2015) and drift does not induce significant linkage-disequilibrium covariance at the whole-genome level. Assortative mating is yet another process, which can induce significant linkage-disequilibrium covariance as the whole-genome level (e.g., Lynch and Walsh 1998; Walsh and Lynch 2018; Rawlik et al. 2020, 2019).

Similarly, population structure and admixture between populations can influence genetic variance and should be addressed in the future. One way to treat population structure would be to partition individuals by sub-population and calculate separate genetic variances and covariances between sub-populations. However, this approach breaks down with admixture. Admixture could be approached by using whole population genome trees with recombination (Kelleher et al. 2019) and labelling individuals and genome segments with originating sub-populations, and expanding the framework into a population analysis of genetic variance. Finally, there is also the classic partitioning of genetic variance between and within families. An interesting line of future research is how different types of directional selection (between or within family and their combination) changes genic and linkage-disequilibrium components of genetic variance.

A final note on genomic analysis is that the proposed framework does not depend on the assumption of Hardy–Weinberg and linkage equilibrium. It is common to see expressions for genetic variance at a locus of the form 2*p*(1 − *p*)*α*^2^, which assumes independent binomial sampling of alleles with probability *p* (Hardy–Weinberg equilibrium). There is an excess of homozygotes over heterozygotes in some breeding programmes, particularly in plant breeding programmes that use inbreeding. In this case, we have a clear deviation from the Hardy–Weinberg equilibrium, and the expression 2*p*(1 − *p*)*α*^2^ will underestimate genetic variance at a locus. To see this consider *p* = 0.5 and *α* = 1, which gives 2*p*(1 − *p*)*α*^2^ = 0.5, but if we only have reference and alternative homozygotes (50% each) the actual variance is doubled due to complete inbreeding (Wright 1931). While there are expressions that involve inbreeding 2*p*(1 − *p*)(1 + *F*)*α*^2^, where 2*p*(1 − *p*)(1 + *F*) is the variance of genotypes under non-random mating, we suggest a simpler straightforward calculation of the variance of genotypes at a locus and multiplying that variance with *α*^2^. Bulmer (1976) was aware of these differences and partitioned genic variance into the value expected under Hardy–Weinberg equilibrium (binomial sampling of alleles) 2*p*(1 − *p*)*α*^2^ and a deviation due to non-random mating 2*p*(1 − *p*)*Fα*^2^.

### Computational aspects

The framework is based on Sorensen et al. (2001), Lehermeier et al. (2017) and Allier et al. (2019) that used the full Bayesian approach and MCMC sampling. We performed our analyses with the full and empirical Bayesian approach and found a good concordance between the two approaches and true values. However, there was a tendency of the empirical Bayesian approach to underestimate uncertainty of inferred genetic variances due to ignoring uncertainty in estimating model variance parameters (Fig. 6). This underestimation is expected, but it seems that the difference is not large, though this will vary between datasets. There are also frequentist approaches that account for the uncertainty of estimating variance components (e.g. Kenward and Roger 1997). The full Bayesian analysis with the marker-based model is not too computationally demanding if the number of markers is not too large (10–50K markers can be handled easily). The full Bayesian analysis can be quite demanding with the genome-based model on individuals if the number of individuals is large, but equivalence with the marker-based model means we can fit one or another model and back-solve desired effects (Stranden and Garrick 2009), as long as we use normal prior distribution for marker effects.

The observation that leading principal components underestimate genic variance (Fig. 7) requires further studies. We expected that increasing the number of leading principal components would reduce the estimation error, which we observed for genetic variance. In contrast, we observed consistent underestimation for genic variance—even with all principal components. Since we had more markers than individuals, this is likely because ‘null’ components would still have some uncertainty in estimation, which we ignored and, therefore, we underestimated genic variance. Methods presented in the supplementary of Listgarten et al. (2012) could be used to correct for this.

### Assumptions

We made two assumptions. First, we assumed a sufficiently dense panel of markers that collectively capture variation at QTL. An insufficient number of markers will deteriorate the ability of the framework to capture genetic variance at and between QTL. Our simulation shows that with a medium marker density, we can estimate genetic variance in a breeding programme accurately. Still, we highlight that we have simulated a simple genetic architecture with randomly located QTL along the genome. Second, we assumed that allele effects are constant over time and across groups of individuals. This assumption is reasonable for estimation in the sense that we used all the available data to estimate average marker effects as accurately as possible and use them across all individuals. However, allele effects can be time-, environment- or population-specific. We tested how our framework fares in such settings by extending the simulation with genotype-by-year interactions. The results have shown that we overestimate genetic and genic variance in those settings, sometimes by as much as 200%. This miss-match is expected because a too simple statistical model can capture too little genetic variation or capture non-genetic effects as genetic and lead to under- or overestimated genetic variances. While model choice is clearly an issue, we note that the proposed framework can be extended to models with effects such as genotype-by-year interactions, for example, by using a model described in Tolhurst et al. (2019). While estimating time-, environment- or population-specific effects could better reflect reality, accurately estimating such effects is challenging. The random regression and time interval approaches (Tsuruta et al. 2004; Hidalgo et al. 2020) have an advantage in this aspect compared to our framework but do not have the flexibility for the genomic analysis of genetic variance. Nuanced estimation of marker effects will likely be more important for breeding programmes that operate in highly variable environments and introgress germplasm from other populations. Still, there will likely be limited data to estimate separate effects accurately. Estimation of background-specific effects is an active research area in genetics with growing datasets across various populations (e.g., Tolhurst et al. 2019; Peterson et al. 2019; van den Berg et al. 2020). Relatedly, we assumed only additive genetic effects. While both theory and data indicate that the average effect of an allele substitution captures most of genetic variance (Hill et al. 2008), recognition of dominance and epistasis is growing (e.g., Hem et al. 2021; Varona et al. 2018; Alves et al. 2019; Legarra et al. 2021). The estimation of non-additive genetic effects and non-additive genetic variances is a very challenging topic. The proposed framework can be expanded to these settings as well and it is a subject of future research.

## Supplementary information

Supplementary Material

## Data Availability

We provide simulation and analysis code at https://github.com/HighlanderLab/llara_additive_genVar.

## References

[CR1] Akdemir D, Sánchez JI (2016). Efficient breeding by genomic mating. Front Genet.

[CR2] Akdemir D, Beavis W, Fritsche-Neto R, Singh AK, Isidro-Sánchez J (2019). Multi-objective optimized genomic breeding strategies for sustainable food improvement. Heredity.

[CR3] Allier A (2019). Assessment of breeding programs sustainability: application of phenotypic and genomic indicators to a north european grain maize program. Theor Appl Genet.

[CR4] Alves FC (2019). Efficient breeding by genomic mating. Plant Methods.

[CR5] Awata LA, Tongoona P, Danquah E, Efie BE, Marchelo-Dragga PW (2018). Common mating designs in agricultural research and their reliability in estimation of genetic parameters. IOSR J Agric Vet Sci.

[CR6] van den Berg I, MacLeod IM, Reich CM, Breen EJ, Pryce JE (2020). Optimizing genomic prediction for australian red dairy cattle. J Dairy Sci.

[CR7] Bernardo R (1994). Prediction of maize single-cross performance using RFLPs and information from related hybrids. Crop Sci.

[CR8] Bernardo R (1996). Best linear unbiased prediction of maize single-cross performance. Crop Sci.

[CR9] Bernardo R (2002) Breeding for quantitative traits in plants, vol. 1. Stemma Press Woodbury

[CR10] Brooks S, Gelman A, Jones G, Meng X-L (2011) Handbook of Markov Chain Monte Carlo. CRC Press

[CR11] Bulmer M (1971). The stability of equilibria under selection. Heredity.

[CR12] Bulmer M (1976). The effect of selection on genetic variability: a simulation study. Genet Res.

[CR13] Burch KS et al. (2021) Partitioning gene-level contributions to complex-trait heritability by allele frequency identifies disease-relevant genes. bioRxiv 10.1016/j.ajhg.2022.02.012 PMC9069080 35271803

[CR14] Crow JF (2010). On epistasis: why it is unimportant in polygenic directional selection. Philos Trans R Soc B Biol Sci.

[CR15] Efron B (1996). Empirical Bayes methods for combining likelihoods. J Am Stat Assoc.

[CR16] Falconer DS, Mackay TF (1996) Introduction to quantitative genetics. Longman 10.1093/genetics/167.4.1529 PMC1471025 15342495

[CR17] Gaynor RC (2017). A two-part strategy for using genomic selection to develop inbred lines. Crop Sci.

[CR18] Gaynor RC, Gorjanc G, Hickey JM (2021) Alphasimr: an r package for breeding program simulation. G3 11:jkaa017. 10.1093/g3journal/jkaa017 10.1093/g3journal/jkaa017 PMC8022926 33704430

[CR19] Gianola D, de los Campos G, Hill WG, Manfredi E, Fernando R (2009). Additive genetic variability and the Bayesian alphabet. Genetics.

[CR20] Gilks WR, Richardson S, Spiegelhalter D (1995) Markov chain Monte Carlo in practice. Chapman and Hall/CRC

[CR21] Gneiting T, Raftery AE (2007). Strictly proper scoring rules, prediction, and estimation. J Am Stat Assoc.

[CR22] González-Diéguez D (2021). Genomic prediction of hybrid crops allows disentangling dominance and epistasis. Genetics.

[CR23] Gorjanc G, Bijma P, Hickey JM (2015). Reliability of pedigree-based and genomic evaluations in selected populations. Genet Sel Evol.

[CR24] Gorjanc G, Gaynor RC, Hickey JM (2018). Optimal cross selection for long-term genetic gain in two-part programs with rapid recurrent genomic selection. Theor Appl Genet.

[CR25] Gorjanc G, Jenko J, Hearne SJ, Hickey JM (2016). Initiating maize pre-breeding programs using genomic selection to harness polygenic variation from landrace populations. BMC Genomics.

[CR26] Gorjanc G, Hickey JM (2019) AlphaBayes: software for genome-wide marker regression along with fixed and random effects. User Manual. University of Edinburgh, UK

[CR27] Hastie T, Tibshirani R (2004). Efficient quadratic regularization for expression arrays. Biostatistics.

[CR28] Hayes BJ, Visscher PM, Goddard ME (2009). Increased accuracy of artificial selection by using the realized relationship matrix. Genet Res.

[CR29] Hem IG, Selle ML, Gorjanc G, Fuglstad G-A, Riebler A (2021). Robust modeling of additive and nonadditive variation with intuitive inclusion of expert knowledge. Genetics.

[CR30] Henderson CR (1976) A simple method for computing the inverse of a numerator relationship matrix used in prediction of breeding values. Biometrics 32:69–83

[CR31] Hidalgo J (2020). Changes in genetic parameters for fitness and growth traits in pigs under genomic selection. J Anim Sci.

[CR32] Hill WG (2016). Is continued genetic improvement of livestock sustainable?. Genetics.

[CR33] Hill WG, Goddard ME, Visscher PM (2008). Data and theory point to mainly additive genetic variance for complex traits. PLoS Genet.

[CR34] Jordan A, Krüger F, Lerch S (2019). Evaluating probabilistic forecasts with scoringRules. J Stat Softw.

[CR35] Kelleher J (2019). Inferring whole-genome histories in large population datasets. Nat Genet.

[CR36] Kennedy B, Schaeffer L, Sorensen D (1988). Genetic properties of animal models. J Dairy Sci.

[CR37] Kenward MG, Roger JH (1997). Small sample inference for fixed effects from restricted maximum likelihood. Biometrics.

[CR38] Legarra A, Garcia-Baccino CA, Wientjes YCJ, Vitezica ZG (2021) The correlation of substitution effects across populations and generations in the presence of non-additive functional gene action. Genetics iyab138 10.1093/genetics/iyab138 PMC8664574 34718531

[CR39] Lehermeier C, de Los Campos G, Wimmer V, Schön C-C (2017). Genomic variance estimates: with or without disequilibrium covariances?. J Anim Breed Genet.

[CR40] Lin L (1989) A concordance correlation coefficient to evaluate reproducibility. Biometrics 45:255–268 2720055

[CR41] Listgarten J (2012). Improved linear mixed models for genome-wide association studies. Nat Methods.

[CR42] de los Campos G, Sorensen D, Gianola D (2015). Genomic heritability: what is it?. PLoS Genet.

[CR43] de los Campos G, Hickey JM, Pong-Wong R, Daetwyler HD, Calus MPL (2013). Whole-genome regression and prediction methods applied to plant and animal breeding. Genetics.

[CR44] Lush J (1937) Animal breeding plans. Iowa State College Press

[CR45] Lynch M, Walsh B (1998) Genetics and analysis of quantitative traits, vol. 1. Sinauer Sunderland, MA

[CR46] Mather K, Jinks JL (2013) Biometrical genetics: the study of continuous variation. Springer

[CR47] Meuwissen T, Hayes B, Goddard M (2001). Prediction of total genetic value using genome-wide dense marker maps. Genetics.

[CR48] Meyer K (1985) Maximum likelihood estimation of variance components for a multivariate mixed model with equal design matrices. Biometrics 41:153–165 4005372

[CR49] Oakey H, Verbyla A, Pitchford W, Cullis B, Kuchel H (2006). Joint modeling of additive and non-additive genetic line effects in single field trials. Theor Appl Genet.

[CR50] Oakey H, Verbyla AP, Cullis BR, Wei X, Pitchford WS (2007). Joint modeling of additive and non-additive (genetic line) effects in multi-environment trials. Theor Appl Genet.

[CR51] Ødegård J, Indahl U, Strandén I, Meuwissen TH (2018). Large-scale genomic prediction using singular value decomposition of the genotype matrix. Genet Sel Evol.

[CR52] Peterson RE (2019). Genome-wide association studies in ancestrally diverse populations: opportunities, methods, pitfalls, and recommendations. Cell.

[CR53] Piepho HP, Möhring J, Melchinger AE, Büchse A (2008). BLUP for phenotypic selection in plant breeding and variety testing. Euphytica.

[CR54] R Core Team (2019) R: a language and environment for statistical computing. R Foundation for Statistical Computing, Vienna, Austria. https://www.R-project.org

[CR55] Rawlik K, Canela-Xandri O, Tenesa A (2019). Indirect assortative mating for human disease and longevity. Heredity.

[CR56] Rawlik K, Canela-Xandri O, Woolliams J, Tenesa A (2020) Snp heritability: what are we estimating? bioRxiv

[CR57] Schreck N, Piepho H-P, Schlather M (2019). Best prediction of the additive genomic variance in random-effects models. Genetics.

[CR58] Selle ML, Steinsland I, Hickey JM, Gorjanc G (2019). Flexible modelling of spatial variation in agricultural field trials with the R package INLA. Theor Appl Genet.

[CR59] Signorell A et al. (2021) Desctools: tools for descriptive statistics. R package version 0.99.42

[CR60] Sorensen D, Kennedy B (1984). Estimation of genetic variances from unselected and selected populations. J Anim Sci.

[CR61] Sorensen D, Fernando R, Gianola D (2001). Inferring the trajectory of genetic variance in the course of artificial selection. Genet Res.

[CR62] Sorensen D, Gianola D (2007) Likelihood, Bayesian, and MCMC methods in quantitative genetics. Springer Science & Business Media

[CR63] Strandén I, Garrick DJ (2009). Technical note: derivation of equivalent computing algorithms for genomic predictions and reliabilities of animal merit. J Dairy Sci.

[CR64] Thompson R (2019). Desert island papers – a life in variance parameter and quantitative genetic parameter estimation reviewed using 16 papers. J Anim Breed Genet.

[CR65] Thompson R, Brotherstone S, White IM (2005). Estimation of quantitative genetic parameters. Philos Trans R Soc B Biol Sci.

[CR66] Tibshirani R (1996). Regression shrinkage and selection via the lasso. J R Stat Soc Series B Methodol.

[CR67] Tolhurst DJ, Mathews KL, Smith AB, Cullis BR (2019). Genomic selection in multi-environment plant breeding trials using a factor analytic linear mixed model. J Anim Breed Genet.

[CR68] Tsuruta S, Misztal I, Lawlor T (2004). Genetic correlations among production, body size, udder, and productive life traits over time in holsteins. J Dairy Sci.

[CR69] Tusell L, Pérez-Rodríguez P, Forni S, Wu X-L, Gianola D (2013). Genome-enabled methods for predicting litter size in pigs: a comparison. Animal.

[CR70] VanRaden PM (2008). Efficient methods to compute genomic predictions. J Dairy Sci.

[CR71] Varona L, Legarra A, Toro MA, Vitezica ZG (2018). Non-additive effects in genomic selection. Front Genet.

[CR72] Walsh B, Lynch M (2018) Evolution and selection of quantitative traits. OUP Oxford

[CR73] Whittaker JC, Thompson R, Denham MC (2000). Marker-assisted selection using ridge regression. Genet Res.

[CR74] Woolliams J, Berg P, Dagnachew B, Meuwissen T (2015). Genetic contributions and their optimization. J Anim Breed Genet.

[CR75] Wright S (1931). Evolution in Mendelian populations. Genetics.

