# Supplementary material for: Temporal and genomic analysis of additive genetic variance in breeding programmes
Source: Heredity (Edinb). 2021 Dec 15;128(1):21–32. doi: 10.1038/s41437-021-00485-y (PMC8733024; doi:10.1038/s41437-021-00485-y)
Supplement: Supplementary file 1 — Supplementary Material [file 41437_2021_485_MOESM1_ESM.pdf]

Temporal and genomic analysis of additive genetic variance in breeding programmes

Letícia A. de C. Lara[1], Ivan Pocrnic[1], Thiago de P. Oliveira[1], R. Chris Gaynor[1], and Gregor Gorjanc[1]

[1]The Roslin Institute and Royal (Dick) School of Veterinary Studies, The University of Edinburgh, Edinburgh, UK

Corresponding author:

Lara, L. A. C.: The Roslin Institute, The University of Edinburgh, Easter Bush Campus, Midlothian, Edinburgh, EH25 9RG, UK. Phone: +44 0131 651 9100. E-mail: leticia.lara.lac@gmail.com

Supplementary Material

Tables

Table S1: Genetic variance partitioned into genic variance and within- and between-chromosome linkage-disequilibrium (LD) covariances by chromosome for **QTL genotypes** (variation of the QTL genotypes) in headrows (HDRW, year 18) and elite yield trial (EYT, year 21); the genetic variance is the sum of genic variance, within-LD and between-LD (see Figure 2)

Chr	HDRW				EYT			
	Genetic	Genic	Within-LD	Between-LD	Genetic	Genic	Within-LD	Between-LD
1	98.2	61.9	36.3	-5.1	91.6	55.4	36.2	40.6
2	49.9	55.3	-5.4	-7.4	65.0	44.5	20.5	-39.2
3	50.5	55.8	-5.3	-15.5	52.6	47.4	5.2	-97.4
4	48.0	52.5	-4.5	-7.9	20.6	51.4	-30.8	14.2
5	49.8	55.6	-5.8	-17.4	41.6	43.0	-1.4	-51.6
6	55.1	60.4	-5.3	1.8	45.2	57.2	-12.0	-58.6
7	53.2	61.9	-8.6	0.1	32.2	54.0	-21.8	40.4
8	84.6	47.7	36.9	7.5	153.0	43.0	110.0	153.2
9	73.8	65.7	8.2	-52.4	47.8	58.3	-10.5	-38.6
10	65.1	57.5	7.6	8.0	104.6	57.2	47.4	-281.4
11	49.6	61.5	-11.9	-1.0	25.6	58.8	-33.1	-14.6
12	40.9	62.4	-21.5	4.4	89.8	51.2	38.6	-82.4
13	48.2	63.4	-15.2	14.0	39.4	49.2	-9.8	9.8
14	68.4	59.2	9.3	14.6	34.6	48.4	-13.8	-1.6
15	50.2	56.8	-6.6	-2.2	55.2	48.2	7.0	69.6
16	86.6	61.6	24.9	-48.8	73.2	50.6	22.6	-35.0
17	65.0	58.5	6.6	29.5	74.0	55.8	18.2	134.6
18	57.0	60.0	-3.0	-10.5	53.0	49.9	3.0	-24.2
19	54.9	60.7	-5.8	4.7	37.4	50.0	-12.6	7.0
20	29.6	58.2	-28.6	8.9	36.4	47.0	-10.6	19.0
21	34.3	58.7	-24.4	-1.5	28.0	53.9	-25.9	-13.2
Sum	1213.1	1235.1[1]	-22.0[2]	-76.2[3]	1200.8	1074.4[1]	126.3[2]	-249.4[3]
Whole-genome[1+2+3]			1136.9				951.4	

Table S2: Genetic variance partitioned into genic variance and within- and between-chromosome linkage-disequilibrium (LD) covariances by chromosome for **true genetic values** (variation of QTL genotypes combined with their true effects) in headrows (HDRW, year 18) and elite yield trial (EYT, year 21); the genetic variance is the sum of genic variance, within-LD and between-LD (see Figure 2)

Chr	HDRW				EYT			
	Genetic	Genic	Within-LD	Between-LD	Genetic	Genic	Within-LD	Between-LD
1	0.0036	0.0039	-0.0003	-0.0010	0.0014	0.0031	-0.0017	-0.0056
2	0.0047	0.0046	0.0001	-0.0020	0.0030	0.0033	-0.0003	-0.0021
3	0.0035	0.0042	-0.0007	0.0011	0.0014	0.0040	-0.0027	0.0028
4	0.0029	0.0039	-0.0010	-0.0002	0.0030	0.0036	-0.0005	0.0017
5	0.0050	0.0037	0.0013	-0.0008	0.0040	0.0027	0.0013	0.0004
6	0.0030	0.0026	0.0004	-0.0017	0.0016	0.0025	-0.0009	0.0004
7	0.0041	0.0041	0.0000	0.0002	0.0042	0.0035	0.0008	-0.0002
8	0.0023	0.0035	-0.0012	-0.0006	0.0031	0.0036	-0.0005	0.0021
9	0.0044	0.0043	0.0001	-0.0002	0.0042	0.0038	0.0004	-0.0040
10	0.0025	0.0033	-0.0008	-0.0003	0.0045	0.0033	0.0013	-0.0075
11	0.0023	0.0037	-0.0014	0.0004	0.0016	0.0035	-0.0019	-0.0052
12	0.0054	0.0043	0.0010	0.0000	0.0048	0.0036	0.0012	-0.0031
13	0.0056	0.0037	0.0019	-0.0005	0.0076	0.0028	0.0048	-0.0087
14	0.0026	0.0045	-0.0019	-0.0004	0.0037	0.0039	-0.0002	-0.0084
15	0.0044	0.0034	0.0010	-0.0004	0.0035	0.0034	0.0001	0.0001
16	0.0058	0.0053	0.0005	-0.0027	0.0082	0.0042	0.0040	-0.0075
17	0.0060	0.0051	0.0009	-0.0019	0.0075	0.0052	0.0022	0.0008
18	0.0038	0.0042	-0.0004	0.0010	0.0034	0.0032	0.0002	0.0003
19	0.0039	0.0038	0.0001	-0.0020	0.0022	0.0030	-0.0007	0.0038
20	0.0033	0.0036	-0.0003	0.0002	0.0009	0.0026	-0.0017	-0.0007
21	0.0030	0.0037	-0.0007	-0.0006	0.0017	0.0033	-0.0016	0.0019
Sum	0.0820	0.0833[1]	-0.0013[2]	-0.0124[3]	0.0756	0.0721[1]	0.0035[2]	-0.0387[3]
Whole-genome[1+2+3]	0.0696				0.0369			

Table S3: Genetic variance partitioned into genic variance and within- and between-chromosome linkage-disequilibrium (LD) covariances by chromosome for **marker genotypes** (variation of marker genotypes) in headrows (HDRW, year 18) and elite yield trial (EYT, year 21); the genetic variance is the sum of genic variance, within-LD and between-LD (see Figure 2)

Chr	HDRW				EYT			
	Genetic	Genic	Within-LD	Between-LD	Genetic	Genic	Within-LD	Between-LD
1	286.9	310.2	-23.3	-156.1	151.2	278.7	-127.4	619.1
2	383.6	288.6	95.0	-151.0	450.4	246.4	204.0	18.8
3	270.1	289.9	-19.8	44.2	435.2	257.4	177.8	829.2
4	371.8	288.4	83.4	125.8	268.0	267.0	1.0	507.2
5	317.5	286.2	31.3	20.6	117.4	211.1	-93.7	-24.0
6	347.0	290.8	56.2	59.1	395.4	278.9	116.5	848.3
7	337.1	311.9	25.2	-172.7	692.8	289.4	403.4	-1021.2
8	340.5	274.2	66.4	-243.9	263.8	221.6	42.2	-1231.2
9	290.1	302.8	-12.6	11.7	133.6	285.6	-151.9	242.4
10	403.9	317.0	86.9	-16.6	473.0	305.2	167.8	816.6
11	192.7	304.2	-111.4	45.9	48.6	290.2	-241.6	-129.7
12	316.0	300.9	15.1	-43.6	230.6	243.5	-13.0	-180.2
13	303.6	294.8	8.8	-175.6	114.6	245.9	-131.4	416.7
14	285.6	315.7	-30.1	34.5	95.6	277.6	-182.0	-346.5
15	221.1	292.8	-71.8	-32.2	319.2	256.8	62.5	25.1
16	396.9	298.3	98.6	-0.2	215.4	248.4	-33.0	213.4
17	322.9	301.3	21.7	-24.8	467.4	283.0	184.3	-1384.9
18	229.8	290.1	-60.3	-32.3	105.2	245.6	-140.5	532.7
19	225.4	307.3	-81.9	48.6	88.2	273.5	-185.4	-16.1
20	404.2	296.3	107.9	-58.4	234.4	245.7	-11.3	175.2
21	205.9	286.7	-80.8	-119.4	146.8	255.4	-108.6	3.1
Sum	6452.6	6248.3^{1}	204.3^{2}	-836.7^{3}	5446.8	5507.0^{1}	-60.2^{2}	914.1^{3}
Whole-genome^{1+2+3}			5615.9				6360.8	

Table S4: Genetic variance partitioned into genic variance and within- and between-chromosome linkage-disequilibrium (LD) covariances by chromosome for **estimated genetic values** (variation of marker genotypes combined with their estimated effects from a statistical model) in headrows (HDRW, year 18) and elite yield trial (EYT, year 21); the genetic variance is the sum of genic variance, within-LD and between-LD (see Figure 2)

Chr	HDRW				EYT			
	Genetic	Genic	Within-LD	Between-LD	Genetic	Genic	Within-LD	Between-LD
1	0.0037	0.0041	-0.0004	0.0003	0.0041	0.0037	0.0004	-0.0029
2	0.0034	0.0038	-0.0004	0.0005	0.0031	0.0033	-0.0002	-0.0009
3	0.0044	0.0039	0.0005	0.0012	0.0040	0.0035	0.0005	-0.0006
4	0.0033	0.0038	-0.0005	-0.0007	0.0035	0.0035	-0.0001	-0.0029
5	0.0044	0.0039	0.0005	-0.0004	0.0030	0.0028	0.0001	-0.0024
6	0.0037	0.0039	-0.0002	-0.0011	0.0027	0.0037	-0.0010	-0.0009
7	0.0037	0.0042	-0.0005	-0.0005	0.0027	0.0039	-0.0011	-0.0016
8	0.0031	0.0037	-0.0006	-0.0004	0.0023	0.0030	-0.0007	-0.0011
9	0.0039	0.0040	-0.0001	0.0004	0.0038	0.0038	0.0000	-0.0021
10	0.0037	0.0042	-0.0005	0.0000	0.0030	0.0041	-0.0011	-0.0018
11	0.0037	0.0040	-0.0003	0.0001	0.0040	0.0039	0.0002	-0.0031
12	0.0041	0.0041	0.0000	-0.0005	0.0038	0.0033	0.0004	-0.0025
13	0.0045	0.0040	0.0005	0.0008	0.0028	0.0033	-0.0005	-0.0011
14	0.0033	0.0042	-0.0009	-0.0005	0.0024	0.0037	-0.0012	-0.0011
15	0.0037	0.0039	-0.0002	-0.0010	0.0023	0.0034	-0.0011	-0.0008
16	0.0040	0.0040	0.0000	-0.0012	0.0031	0.0033	-0.0002	-0.0027
17	0.0040	0.0041	-0.0001	0.0003	0.0034	0.0038	-0.0004	-0.0017
18	0.0035	0.0039	-0.0003	-0.0004	0.0025	0.0033	-0.0008	-0.0007
19	0.0038	0.0041	-0.0003	-0.0004	0.0030	0.0037	-0.0007	-0.0006
20	0.0038	0.0040	-0.0002	0.0005	0.0031	0.0033	-0.0002	-0.0022
21	0.0034	0.0038	-0.0004	-0.0006	0.0030	0.0034	-0.0004	-0.0010
Sum	0.0791	0.0836[1]	-0.0045[2]	-0.0037[3]	0.0655	0.0736[1]	-0.0081[2]	-0.0348[3]
Whole-genome[1+2+3]	0.0754				0.0307			

Table S5: Concordance correlation coefficient (CCC), Pearson correlation coefficient (r), and bias corrector factor (C_b) between estimated and true genetic and genic variances across replicates considering absence/presence of genotype-by-year interaction (noGxY/GxY) and number of QTL

Interaction	Number of QTL	Genetic variance			Genic variance		
		CCC (lower ; upper)	r	C_b	CCC (lower ; upper)	r	C_b
noGxY	210	0.81 (0.68 ; 0.90)	0.86	0.94	0.77 (0.66 ; 0.84)	0.97	0.79
noGxY	2100	0.86 (0.75 ; 0.92)	0.89	0.96	0.84 (0.75 ; 0.89)	1.00	0.84
noGxY	21000	0.84 (0.72 ; 0.91)	0.88	0.94	0.86 (0.78 ; 0.91)	1.00	0.86
GxY	210	0.29 (0.18 ; 0.40)	0.65	0.41	0.29 (0.23 ; 0.35)	0.97	0.30
GxY	2100	0.43 (0.27 ; 0.56)	0.74	0.57	0.42 (0.33 ; 0.51)	1.00	0.42
GxY	21000	0.30 (0.17 ; 0.41)	0.74	0.39	0.26 (0.18 ; 0.33)	1.00	0.26

Figures

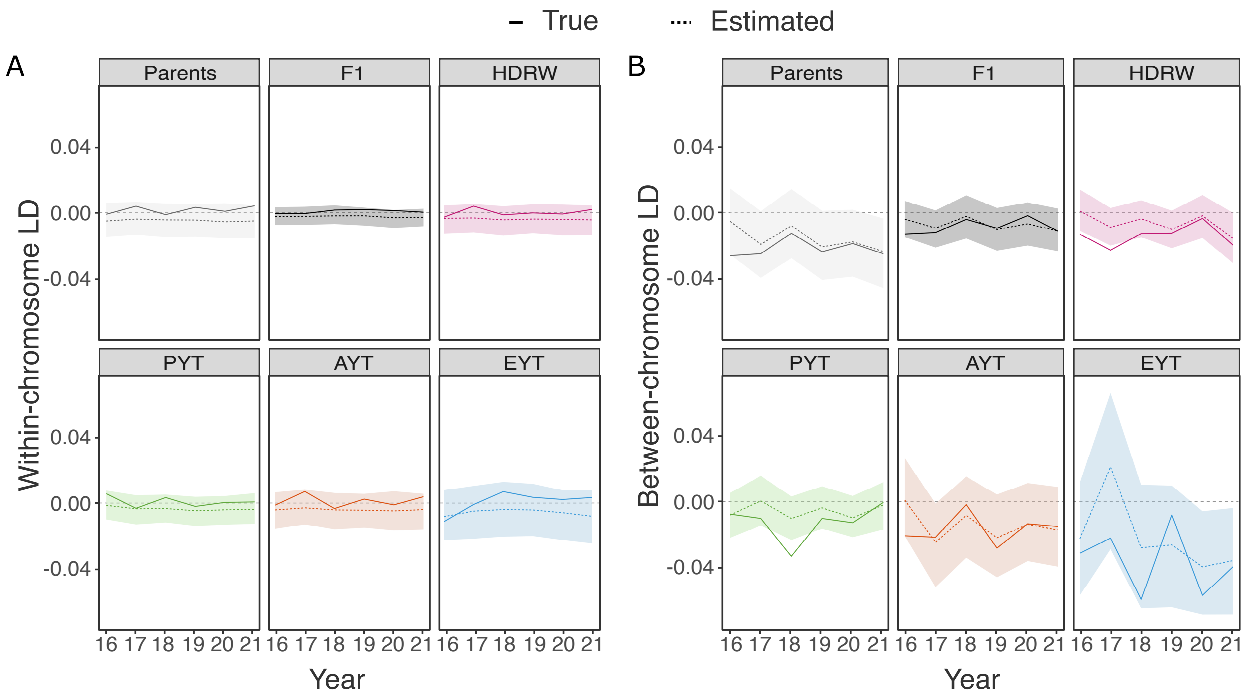

Figure S1: Temporal trend in within-chromosome (A) and between-chromosome (B) linkage disequilibrium estimated with the full Bayesian approach for parents, F_1 progeny (F1), headrows (HDRW), preliminary yield trial (PYT), advanced yield trial (AYT), and elite yield trial (EYT); solid lines denote the true value, dashed lines denote posterior means and polygons depict 95% posterior quantiles

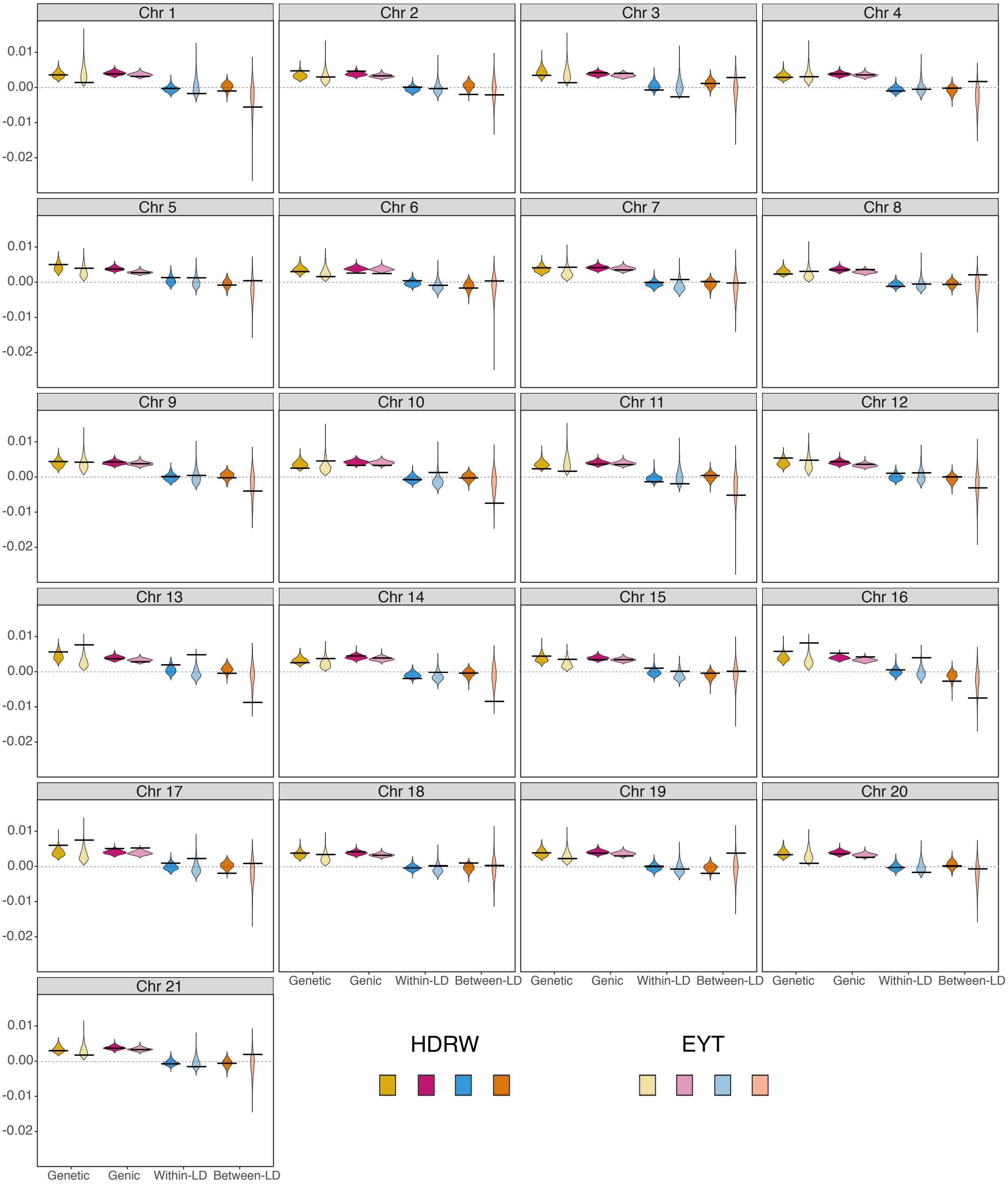

Figure S2: Genetic and genic variances, and within- and between-chromosome linkage disequilibrium (LD) covariances by chromosome with the full Bayesian approach for headrows (HDRW, year 18) and elite yield trial (EYT, year 21) (see Figure 2); black lines denote true values and violins depict posterior distributions

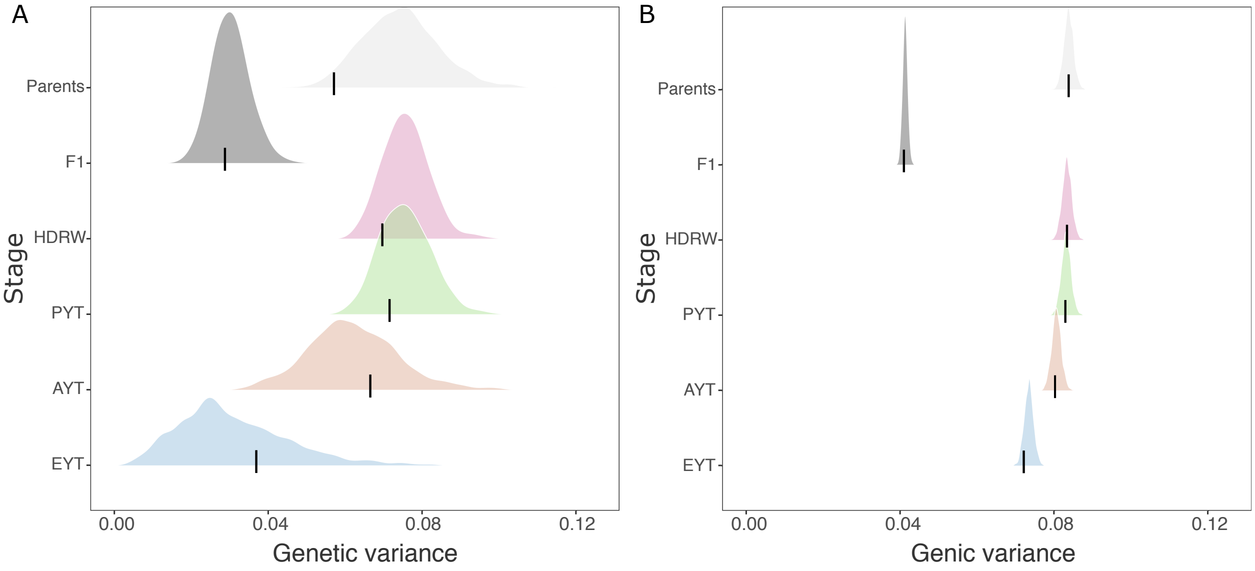

Figure S3: Genetic (A) and genic (B) variance estimated with the empirical Bayesian approach for parents in year 16, F_1 progeny (F1) in year 17, headrows (HDRW) in year 18, preliminary yield trial (PYT) in year 19, advanced yield trial (AYT) in year 20, and elite yield trial (EYT) in year 21; black lines denote the true values and densities depict posterior distributions

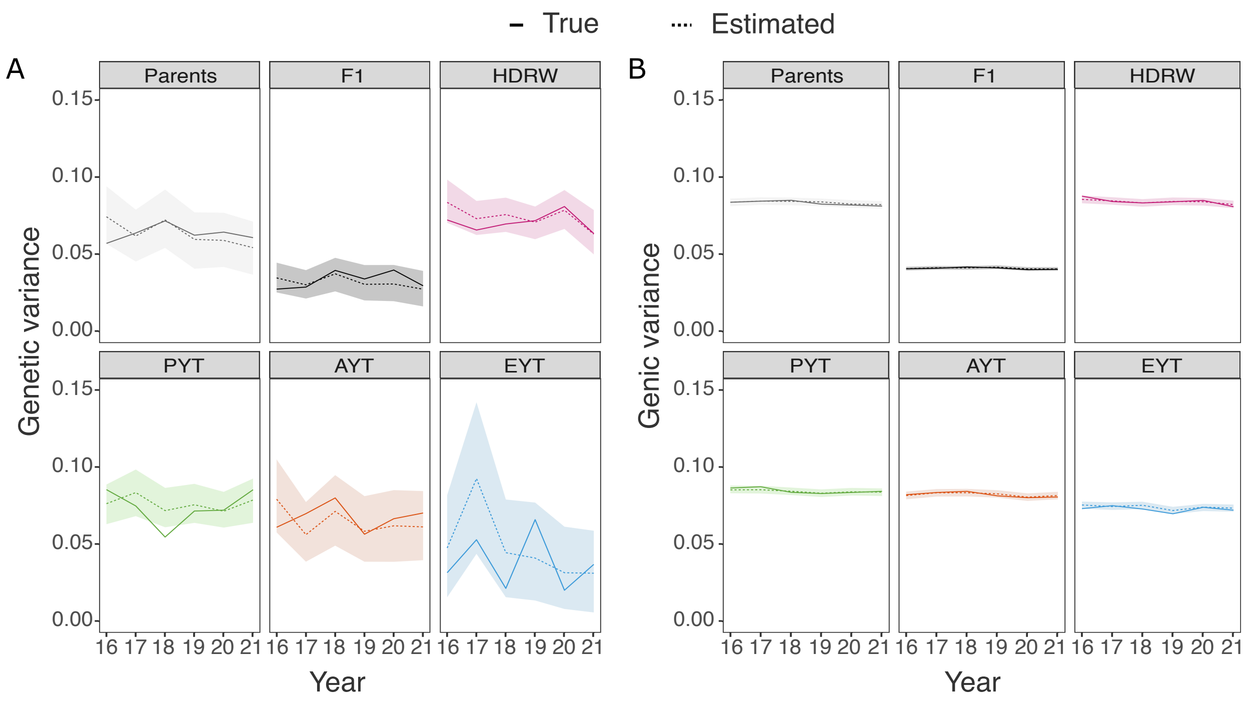

Figure S4: Temporal trend in genetic (A) and genic (B) variance estimated with the empirical Bayesian approach for parents, F_1 progeny (F1), headrows (HDRW), preliminary yield trial (PYT), advanced yield trial (AYT), and elite yield trial (EYT); solid lines denote the true value, dashed lines denote posterior means and polygons depict 95% posterior quantiles

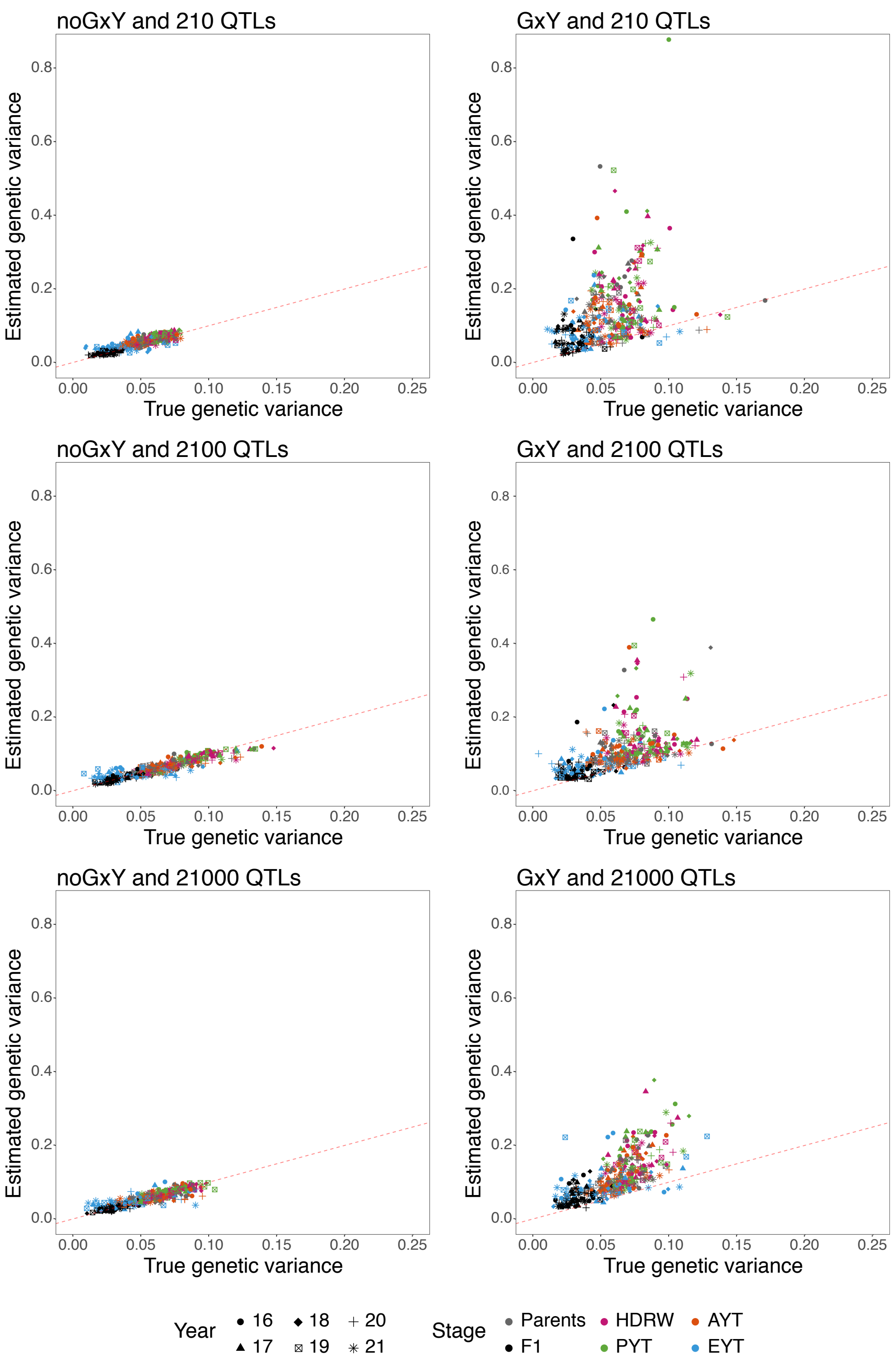

Figure S5: Estimated genetic variance (posterior mean from the full Bayesian approach) versus true genetic variance for each breeding stage, year and replicate considering absence/presence of genotype-by-year interaction (noGxY/GxY) and number of QTL; equal values are represented by the dashed red line

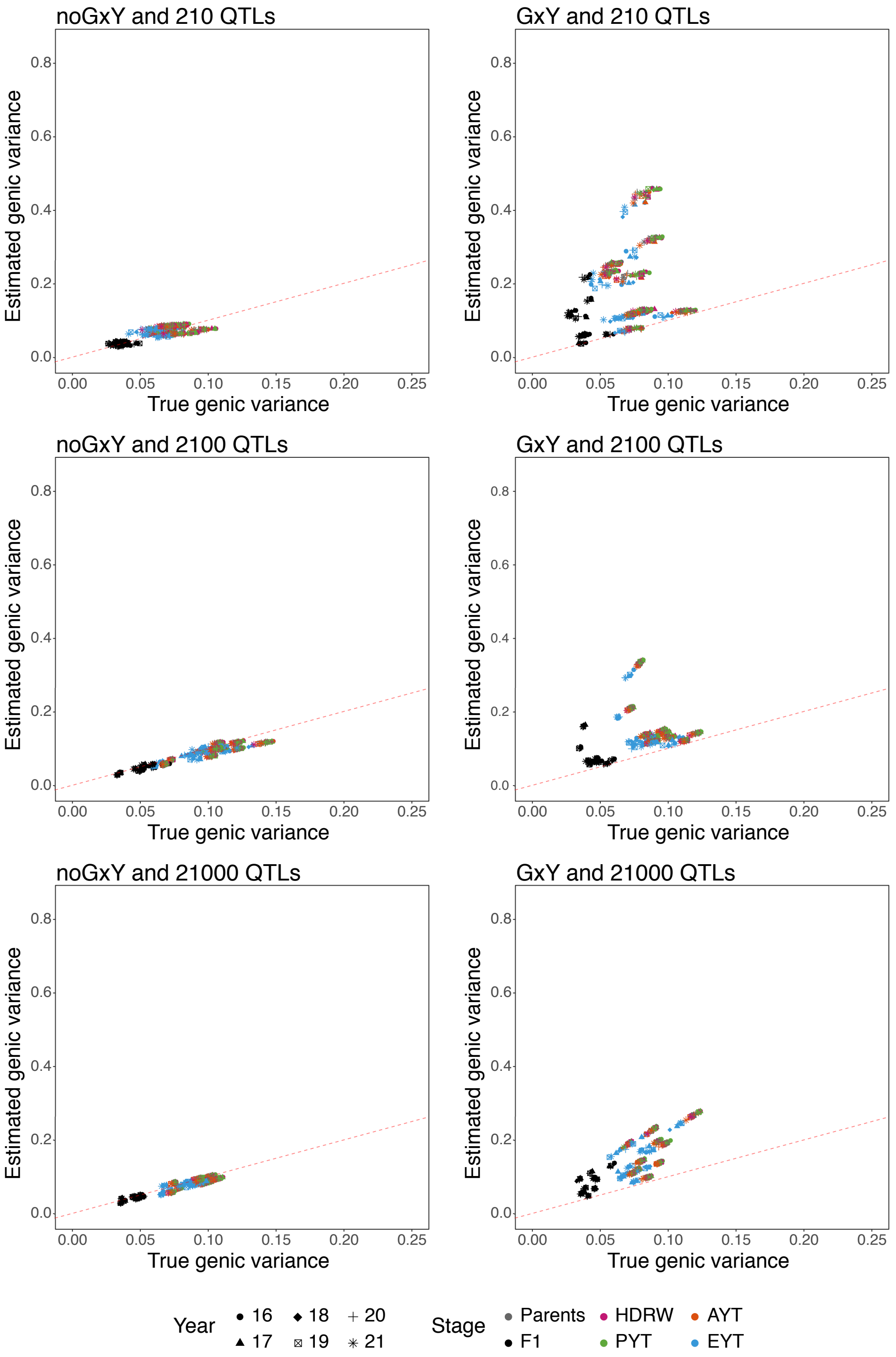

Figure S6: Estimated genic variance (posterior mean from the full Bayesian approach) versus true genic variance for each breeding stage, year and replicate considering absence/presence of genotype-by-year interaction (noGxY/GxY) and number of QTL; equal values are represented by the dashed red line
